# Feasibility and effectiveness of electronic vs. paper partograph on improving birth outcomes: A prospective crossover study design

**DOI:** 10.1371/journal.pone.0222314

**Published:** 2019-10-07

**Authors:** Aminur Rahman, Tahmina Begum, Fatema Ashraf, Sadika Akhter, Dewan Md. Emdadul Hoque, Tarun Kanti Ghosh, Monjur Rahman, Jelle Stekelenburg, Sumon Kumar Das, Parveen Fatima, Iqbal Anwar

**Affiliations:** 1 Health Systems and Population Studies Division, International Centre for Diarrhoeal Disease Research Bangladesh (icddr,b), Dhaka, Bangladesh; 2 Department of Obstetrics and Gynaecology, Shaheed Suhrawardi Medical College & Hospital, Dhaka, Bangladesh; 3 Maternal and Child Health Division, icddr,b, Dhaka, Bangladesh; 4 Department of Obstetrics and Gynaecology, Kushtia Medical College & Hospital, Kushtia, Bangladesh; 5 Department of Health Sciences, Global Health, University Medical Centre/University of Groningen, Groningen, The Netherlands; 6 Department of Obstetrics and Gynaecology, Leeuwarden Medical Centre, Leeuwarden, The Netherlands; 7 Clinical and Nutrition Sciences Division, icddr,b, Dhaka, Bangladesh; 8 Department of Obstetrics and Gynaecology, Bangabandhu Sheikh Mujib Medical University, Dhaka, Bangladesh; Karolinska Institutet, SWEDEN

## Abstract

**Background:**

The partograph has been endorsed by World Health Organization (WHO) since 1994 which presents an algorithm for assessing maternal and foetal conditions and labor progression. Monitoring labour with a partograph can reduce adverse pregnancy outcomes such as prolonged labor, emergency C-sections, birth asphyxia and stillbirths. However, partograph use is still very low, particularly in low and middle income countries (LMICs). In Bangladesh the reported partograph user rate varies from 1.4% to 33.0%. Recently, an electronic version of the partograph, with the provision of online data entry and user aid for emergency clinical support, has been tested successfully in different settings. With this proven evidence, we conducted and operations research to test the feasibility and effectiveness of implementing an e-partograph, for the first time, in 2 public hospitals in Bangladesh

**Methods:**

We followed a prospective crossover design. Two secondary level referral hospitals, Jessore and Kushtia District Hospital (DH) were the study sites. All pregnant women who delivered in the study hospitals were the study participants. All nurse-midwives working in the labor ward of study hospitals were trained on appropriate use of both types of partograph along with standard labour management guidelines. Collected quantitative data was analyzed using SPSS 23 statistical software. Discrete variables were expressed as percentages and presented as frequency distribution and cross tabulations. Chi square tests were employed to test the association between exposure and outcome variables. Potential confounding factors were adjusted using multivariate binary logistic regression methods. Ethical approval was obtained from the institutional review board of the International Centre for Diarrheal Disease Research, Bangladesh (icddr,b).

**Findings:**

In total 2918 deliveries were conducted at Jessore DH and 2312 at Kushtia DH during one-year study period. Of them, 1012 (506 in each facility) deliveries were monitored using partograph (paper or electronic). The trends of facility based C-section rates was downwards in both the hospitals; 43% to 37% in Jessore and from 36% to 25% in Kushtia Hospital. There was a significant reduction of prolonged labour with e-partograph use. In Kushtia DH, the prolonged labour rate was 42% during phase 1 with the paper version which came down to 29% during phase-2 with the e-partograph use. The similar result was observed in Jessore DH where the prolonged labour rate reduced to 7% with paper partograph from the reported 30% prolonged labour with e-partograph. The e-partograph user rate was higher than the paper partograph during both phases (phase 1: 3.31, CI: 2.04–5.38, p < .001 and in phase 2: 15.20 CI: 6.36–36.33, p < .001) after adjusting for maternal age, parity, gestational age, religion, mother’s education, husband’s education, and fetal sex

**Conclusion:**

The partograph user rate has significantly improved with the e- partograph and was associated with an overall reduction in cesarean births. Use of the e-partograph was also associated with reduced rates of prolonged labour. This study has added to the growing body of evidence on the positive impact of e-partograph use. We recommend implementing e-partograph intervention at scale in both public and private hospitals in Bangladesh.

**Trial registration:**

ClinicalTrials.gov NCT03509103.

## Introduction

Annually, an estimated 303,000 maternal and 3.1 million neonatal deaths occur globally [1]. Almost 99% of these deaths are from low- and middle-income countries where women frequently lack access to quality perinatal care [2, 3]. A large proportion of maternal deaths follow prolonged labour resulting in obstructed labour, maternal dehydration, rupture uterus, obstructed fistula, and postpartum hemorrhage (PPH)[4]. Prolonged or obstructed labour is risky for newborn as well as they may cause birth asphyxia, brain damage, infection and death during the neonatal period or later[5]. Obstructed labour with or without rupture uterus ranks among the five major causes of maternal deaths in almost all LMICs, although its relative contribution varies from region to region[6]. Early detection of abnormal labor progression, along with prevention of prolonged labour, helps in reducing maternal and perinatal mortality. Now, the partograph is considered as a unique tool for effective monitoring of labour and identifying women in need of an emergency obstetric interventions [7].

The partograph, a framework for assessing maternal condition, foetal condition and labour progression, dates back to the 1950s. The concept of the partograph and its labour curve was first conceived by E.A. Friedman, an obstetrician [8]. Later on in 1972, R.H. Philpott incorporated alert lines and action lines upon Friedman’s partograph which allowed the partograph to serve as a practical tool for recording all intrapartum observations, and identifying instances of increased risk [9].

In 1994, the World Health Organization (WHO) has extensively tested the efficacy of the partograph and established the scientific basis and rationale for its widespread use in the prevention of prolonged labour [10, 11]. The study conducted by the WHO in South East Asian countries reported that using the partograph as a labour monitoring tool, could reduce prolonged labour from 6.4% to 3.4%, labour augmentation from 20.7% to 9.1%, emergency C-sections rate from 9.9% to 8.3%, and stillbirths rate from 0.5% to 0.3% [12]

Although the outcomes have been positive, there has been difficulty in maximizing utility of the partograph. In a study conducted in Cameroon, it was found that only 30% of respondents had any knowledge about the partograph despite its life saving capabilities [13]. In contrast, another study conducted in Ethiopia regarding knowledge and attitudes on partograph use, has documented some positive results. For example, 70% of the studied obstetric care physicians had a good level of knowledge and 84% showed favorable attitudes, and about 72% agreed that the partograph use is important and effective in decreasing maternal deaths. Despite these positive statistics, partograph use is still very low across the world [14].

In Bangladesh, the use of the partograph is recommended for all institutional deliveries. However, the utilization rate, like many other LMICs, is very low. The highest partograph user rate in Bangladesh was reported at 33%, in a facility based study conducted in two rural districts [15]. Another 24 district need-assessment study reported that partograph user rate was only 3% in institutional settings [16]. A study conducted among 38 nurse/midwives working in the Emergency Obstetric and Newborn Care units in 12 EmONC facilities in six different geographical divisions revealed that 79% of the respondents know what information needs to be recorded on a partograph, however less than 50% of them were using the tool [17]. This scenario clearly depicts that the lack of knowledge is not the only factor for the under-utilization of the partograph in Bangladesh.

However, higher user rate cannot guarantee the correct use of the partograph. For example one study conducted in Kenya found that the partograph user rate was 88%; however only 24% of the pantographs were filled out correctly [7]. In addition to improper knowledge, several other factors have been documented as cause of inadequate or improper use of partograph including: resistance from service providers who consider it an administrative tool, absence of uniform protocols, inadequate training of service providers, a lack of support from the system, and insufficient supply of paper partograph.[18–20]. To combat all these issues, digital technologies were proposed and tested in different settings. For instance, a brief trial was run in Kenya to compare the use of digital partograph to those made of paper. A phone app was designed [21, 22] incorporating all features of the paper partograph with the added bonus of the app being able to monitor the progression of labor in real time. This small-scale initiative has created momentum for the use of the electronic partograph. The WHO looked at this model and developed the ‘maternal health assessment and planning for scale (MAPS)’ initiative to scale up e-partograph to increase its use all over the world [23] Moreover, the John Hopkins Program for International Education in Gynecology and Obstetrics (Jhpiego) tested three e-partograph implementation mechanisms, which included an Android tablet application, a digital clipboard system, and a custom hardware solution. However, none of these technologies were successful in improving partograph user rate. [24]

E-partograph is expected to promote consistent and correct use of the tool. However, scientific evidence is required to convince policy program mangers to scale it up, particularly in low resource settings. With this vision, we conducted an operations research to test the feasibility and effectiveness of digital partograph in two selected district hospitals in Bangladesh.

## Methods and materials

### Study design, site and duration

A prospective, follow-up crossover study design was used. Two secondary level referral hospitals in Jessore and Kushtia districts were selected as study sites. The selected facilities were similar in terms of infrastructure, human resources, and service delivery scenarios to typical district hospitals and were thus representative of secondary level public hospitals in Bangladesh. However, there were some differences between two study hospitals in regards to facility based C-section rates and stillbirth rates. The crossover study design was adopted to adjust the effect of this non-similarity upon study outcomes. The same facility was served as its own control at different point in time [25, 26]. We conducted the study in two phases (February 2015- July 2016) with an intervening pause period. During the first phase (June-July 2015), the E-partograph was used in Kushtia district hospital and a paper based partograph in Jessore district hospital. Both of these hospitals were being crossed to alternate partograph tool after the pause period during the second phase of the study (October-December 2015).

All nurse-midwives posted in obstetric wards in each study district hospital were the users who conduct all deliveries in these hospitals. At the start of the project, a two-day training was organized for the nurse-midwives so that they become acquainted with the respective partograph E- or paper-partograph that they would be using during the first phase of the study. Another training session was also arranged during the pause period. Clinical parameters recorded in the partograph were validated by project research assistants with midwifery qualifications who also observed labor progression and deliveries.

### Sample selection

**Fig 1:**
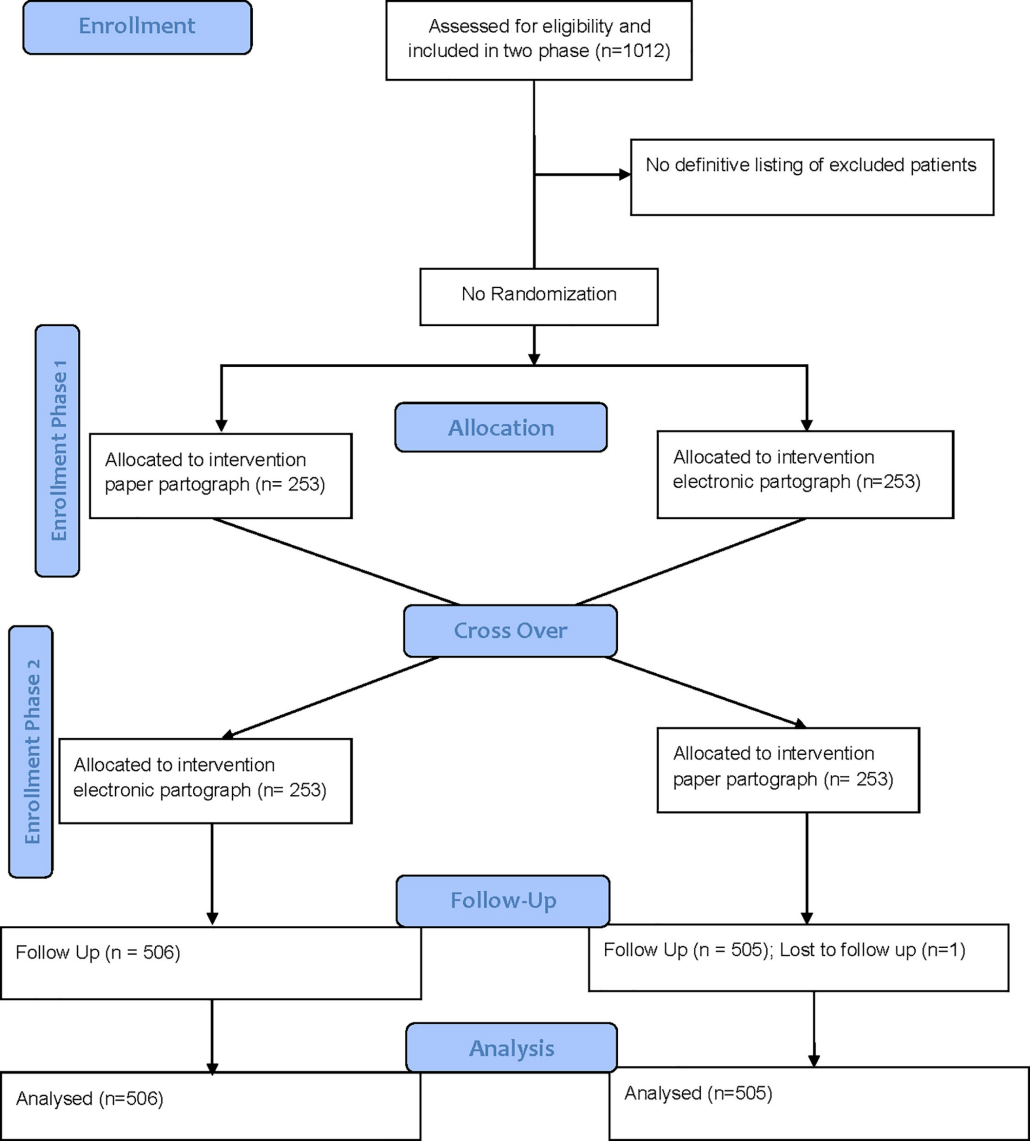
Consort flow chart.

Minimum sample size were calculated based on (1) existing prevalence of partograph use (17.1%) [15]), and (2) adverse delivery outcome rates related to partograph use such as incidence of birth asphyxia (22%) [27, 28]) and prolonged labour (7% [29]). Sample size calculations also took into account the refusal rate (10%). Using 36% as prevalence of partograph use, 11% as prevalence of birth asphyxia and 2.8% as prevalence of prolonged labour, the minimum sample size required were, 105, 216 and 506 respectively at 80% power, 5% significance level. Thus the final sample size in each district hospital was taken as 506; half of the sample (253) before and the other half (253) after applying the crossover design. Field data collection continued for 12 months and the data collectors tried to capture all deliveries until the required minimum sample size was achieved (Fig 1).

### Inclusion criteria

Inclusion criteria for the study were women with spontaneous labour in the first stage of labour with cervical dilatation in between 4–7 centimeters, singleton pregnancy, gestation of at least 37 completed weeks, cephalic presentations, and no additional complications.

### Exclusion criteria

Exclusion criteria for the study were women with ante partum hemorrhage, eclampsia, elective caesarean section, and induced labour.

### Study procedure and data collection

An expert obstetrician, who is also a study investigator, provided training to the nurse-midwives working in the obstetric wards of the study hospitals. The basics for the paper and electronic partograph were essentially the same, so the training methods were not vastly different. We have used WHO partograph both for paper and electronic version. For the e-partograph, a junior programmer was available by mobile phone, round-the-clock, to provide any instant troubleshooting or feedback. In addition, the Android Tablet was used for implementing the digital partograph study. Participants were also introduced to varying inconsistencies and errors they might face when utilizing the e-partograph. The research assistants became expert users of the e-partograph and facilitated the use for the weak users.

#### E-partograph

The electronic version of the partograph is a state-of-the-art application that could be accessed through smart phone or tablet pc or computer device. The application’s user interface (UI) is segmented. Thus the users have to concentrate only on a single portion of the partograph one at a time that lessen the existing complexity of using paper-based partograph. In our study, the -partograph application’s user interface was used in Android programming language for smart tabs, and in ASP.net with C# language for personal computers. There was in build mechanism in the e-partograph software to give a red signal for any maternal or fetal abnormality during active phase of labour. For example crossing the alert line, high BP, poor uterine contraction or altered fetal heart rate it also provide pop up window for interpreting findings. The application had options to save the data both in local storage and in remote central database storage concurrently. Local storage contains data for temporarily; the remote server contains the data permanently which made the partograph information searchable at any time and place. This application allow partograph data to be monitored remotely.

The existing knowledge on how to use the paper and e-partograph was assessed for each participant before the start of the training to understand the skill status of the individuals. The training consisted of two days of intensive hands on training as well as a refresher course that was organized six months after the first training to ensure that participants would not forget the information. In order to measure user rates, rates of birth asphyxia, and prolonged labour, record reviews generated by the partograph were collected by study research assistants and analyzed by study investigators.

### Data analysis

Quantitative data was analyzed using SPSS 23. The outcome variables were use of the partograph and delivery outcomes (prolonged labour and birth asphyxia). The independent variables covered maternal demographic and obstetric variables along with fetal characteristics as well as type of partograph and facility. Partograph was considered done when it was correctly used for labour monitoring among the delivered women who fall under inclusion criteria. Prolonged labour was defined as labour extending more than 12 hours and birth asphyxia was defined as an APGAR score less than 7 on the 5th minute after delivery. Discrete variables were expressed as percentages and presented as frequency tables and cross tabulations. Chi square (χ2) tests were employed to test the association between dependent and independent variables. Statistical significance was defined as p-values of <0.05. McNemar test has been applied to assess the significance of (increasing or decreasing) trend of c-section rate over 12 month time period for both the hospitals. Binary logistic regression was used to compare user rate of both types of partograph separately for both the study health facilities after adjusting for the other covariates. Potential confounding factors were adjusted using binary logistic regression for analysis of uptake.

### Ethical assurance

The Research Review Committee (RRC) of the International Centre for Diarrheal Disease Research, Bangladesh (icddr, b) approved the technical part of the proposed study. Then the Ethical Review Committee (ERC) of icddr, b who oversees the protection of human rights, approved the study. In addition, informed written consent was obtained from the hospital administration, health workers, and the laboring mothers. In case of minor pregnant women, the assent was sought accordingly.

The study was not registered as clinical trial before data collection for the nature of the design (prospective cross over design) and it was not mandatory for the IRB approval. However, “The authors confirm that all ongoing and related trials for this intervention are registered”.

The Institutional review Board approved research protocol and one relevant critical Appraisal checklist (TREND checklist) are added as supplementary information S1 Table & S2 Table respectively.

## Results

**Fig 2:**
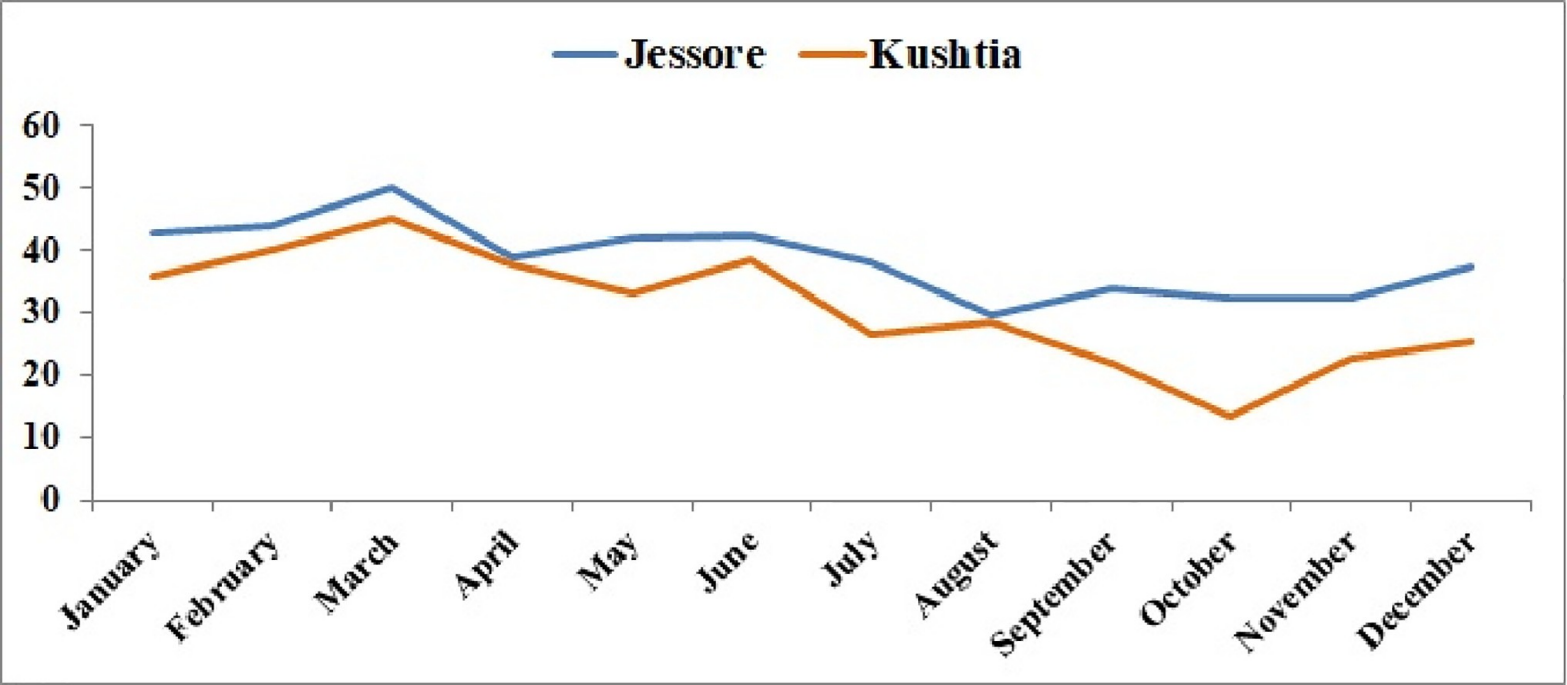
Delivery caesarean section by month in Jessore and Kushtia districts hospital.

During one year study period, a total of 5230 deliveries were conducted in two study hospitals: 2918 in Jessore district hospital and 2312 in Kushtia district hospital. The majority of these deliveries were normal vaginal deliveries; however, the rates of caesarean delivery showed some downwards trends during the study period in both the hospitals. In Jessore DH, facility based the C-section rate was 43% in January which subsequently reduced to 37% in the month of December (p-value = 0.001 < 0.05) (Fig 2).

Similarly, the C-section rate in Kushtia DH reduced from 36% to 25% during same period of time (p-value = 0.001 < 0.05) (Fig 2). The C-section rates started declining in the month of June, when the project started to monitor partograph user rates as well as completeness of partograph through direct observation.

A total of 506 deliveries were directly observed in two study DHs using partograph. The selection of delivery cases were according to inclusion and exclusion criteria and first-come first-enrollment basis. The demographic and obstetric characteristics of the delivering women, along with their fetal characteristics, are presented in the Tables 1 and 2 that shows the mothers in Jessore belong to Muslim religion (p < .002) and with lower parity (p<0.001) compared to mothers of Kushtia. There were no differences in regards to fetal characteristics. (Table 2)

**Table 1:** Maternal demographic and obstetric characteristics (Religion and education has missing value).

	Jessore (N = 506) N (%)	Kushtia (N = 505) N (%)	P-Value
**Maternal Age (years)**			
<19	53(10.47)	44(8.71)	0.49
20–29	354(69.96)	351(69.50)	
≥30	99(19.57)	110(21.78)	
**Religion**			
Muslim	474(94.99)	496(98.41)	0.002
Non-Muslim	25(5.01)	8(1.59)	
**Parity**			
0	286(56.52)	227(44.95)	0.001
1	128(25.30)	174(34.46)	
>2	92(18.18)	104(20.59)	
**Maternal education**			
Primary complete	108(24.00)	133(28.90)	0.201
Secondary complete	281(62.44)	277(60.22)	
Higher secondary and above	61(13.56)	51(11.09)	
**Husband education**			
Primary complete	86(24.16)	97(28.61)	0.119
Secondary complete	199(55.90)	163(48.08)	
Higher secondary and above	71(19.94)	79(23.30)	

**Table 2:** Fetal characteristics.

	Jessore (N = 506) N (%)	Kushtia (N = 505) N (%)	P-Value
**Birth Outcome**			
Live Birth	491(97.04)	483(95.64)	0.239
Stillbirth	15(2.96)	22(4.36)	
**Sex of Child**			
Male	237(46.93)	253(50.20)	0.299

**Fig 3:**
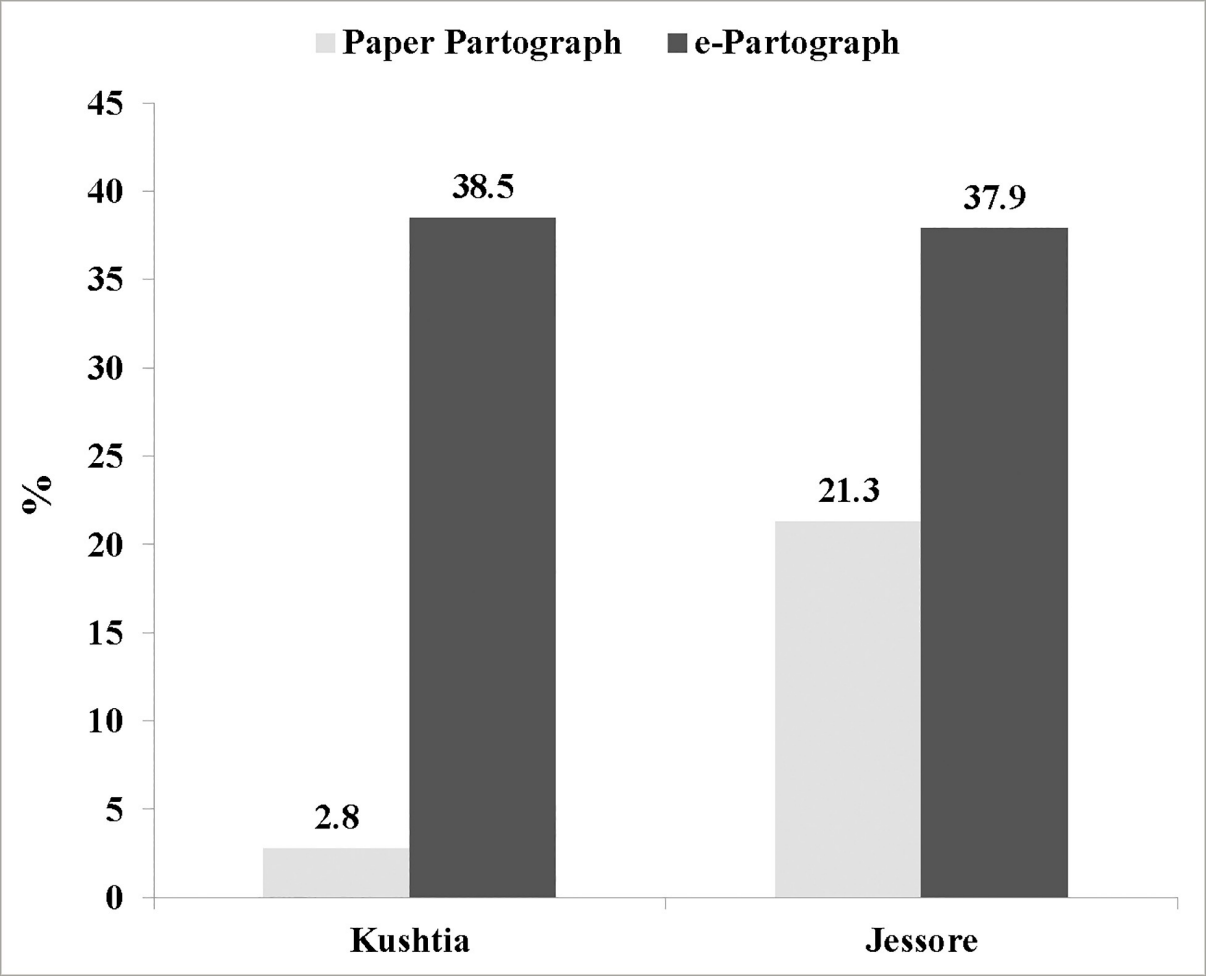
Partograph user rate among two study facilities during two phases.

Due to cross over nature of the study Kushtia DH was 1^st^ exposed to e-partograph with the recorded user rate of 38.5% during phase 1 and then the paper partograph user rate was reported as 2.8% during phase 2 (p<0.001). On the other hand, Jessore used paper-partograph during phase 1 and E-partograph during phase 2 and the user rate was 21.3% during phase 1 and 37.9% during phase 2 of the study (p<0.001) (Fig 3).

In bivariate analysis, we examined the association between partograph use and prevalence of prolonged labour and birth asphyxia at 5^th^ minute of delivery outcome in both the study sites. The analyses were stratified by phase of the study to look into the influence of partograph type on the prolonged labour and birth asphyxia (Table 3). The rates of reduction of prolonged labour were more with the use of e-partograph than paper partograph during both the phases except in Kushtia DH. In Kushtia DH, the prolonged labour rate reduced from 30% during phase-1 (electronic) to 7% during phase-2 (paper). While in Jessore DH prevalence of prolonged labour reduced from 42% during phase 1 (paper) to 29.6% during phase 2 (electronic) (Table 3).

**Table 3:** Prolonged labor & birth asphyxia rate by the type of the partograph and by the phases of the study.

	Jessore			Kushtia		
	Type of Partograph		P-Value	Type of Partograph		P-Value
	Paper (Phase1)	Electronic (Phase2)		Paper (Phase2)	Electronic (Phase1)	
**Prolonged Labor**						
	N = 253 (n %)	N = 253 (n %)		N = 253 (n %)	N = 253 (n %)	
**Yes**	107 (42.3)	75 (29.6)	0.004*	18 (7.1)	77 (30.4)	< .001*
**Birth Asphyxia**						
	N = 246 (n %)	N = 245 (n %)		N = 243 (n%)	N = 241 (n %)	
**Yes**	28 (11.4)	26 (10.6)	0.785	1 (0.4)	2 (0.8)	0.558

*statistically significant at 95% confidence interval

Among all deliveries observed, more than 90% took place successfully before reaching to the alert line which indicates that with successful intervention with partograph on time could reduce the prevalence of complicated deliveries. In this regards, the e-partograph was more effective in identifying poor progression of labour than the paper partograph. (Table 4).

**Table 4:** Position of cervical graph by the type of partograph.

	Paper Partograph (N = 55) n (%)	e-Partograph (N = 190) n (%)	P value
Left to alert line	51(92.73)	181(95.26)	0.46
Between alert and action line	3(5.45)	9(4.74)	0.828
Right to action line	1 (1.82)	0(0)	0.063

Multivariate logistic regression analysis was undertaken to determine the effect of both types of partograph upon user rates. Findings suggest that with use of e-partograph, user rates increases more than with use of paper partograph which is true for both phases the study. The e-partograph user rate was three times higher (OR 3.31, 95% CI: 2.04–5.38; p< 0.001*) than user rate with paper partograph in Phase 1 and 15 times higher in Phase 2 after adjusting for maternal age, parity, gestational age, religion, mother’s education, husband’s education, and fetal sex. (Table 5)

**Table 5:** Regression analysis showing user rate of two partograph types.

*By Facility	Partograph Usages	
	E-Partograph	Paper Partograph
	(OR, 95% CI, P-Value)	Reference
**Phase1**	3.31, CI: 2.04–5.38, p< 0.001*	1
**Phase 2**	15.20, CI: 6.36–36.33, p< 0.001*	1

*Values are adjusted for maternal age, parity, gestational age, religion, mother’s education, husband’s education and fetal sex.

## Discussion

Our study clearly showed that the user rate of the e-partograph is significantly higher than the paper partograph during both the phases; (38% vs. 21.3% on 1^st^ phase and 38% vs. 2.8% on 2^nd^ phase). This finding could be explained more confidently from regression results which showed that partograph use was increased three times during phase I and fifteen-times during phase II when nurses used e-partograph. The influence of confounding factors such as difference in health facility type and variation of provider’s skill and attitude on plotting partograph could be controlled through the cross- over design as the electronic version was equally being used during both the phases. However, there was variation in the use of paper partograph among two study health facilities, much lower in Kushtia district hospital (2.8%) in comparison to Jessore District hospital (21.3%). Since Kushtia hospital was exposed to e–partograph during the 1^st^ phase of the study, staff members were continuing their same practices even at the 2^nd^ phase when they were supposed to use paper partograph.

The higher use of e-partograph observed in this study resulted from some focused interventions. The barriers of partograph use particularly in low resource setting documented under the global fistula project [18–20] were taken into account while designing the intervention packages for this study. Poor knowledge on plotting partograph was mitigated through training & refreshers training for the nurse-midwives by national and local experts. The study nurse-midwives were trained on appropriate use of the partograph, both for the electronic and paper partograph, and were instructed to follow a single labour management protocol. The constraints related to logistics were mitigated through regular supply of paper partograph and internet connectivity to the computer tablet. The addition of the electronic version of partograph provided flexibility in supervision, as it allowed the authority (hospital superintendent, head of obstetric unit and the duty doctors) to make an instant online query about the use of partograph. The study team worked to reduce resistance to the partograph use by educating providers on the clinical usefulness of the tool. As a result, health care providers started to use the partograph as decision making tool instead of an administrative task.[18, 19] The increased user rate with electronic partograph has also been reported in other settings. Save the Children International Kenya has piloted a study in 15 health facilities in Bungoma County, Kenya with a mobile-based partograph system intervention. The Kenyan study could ensure 100% correct use of partograph and the labour complications identified through the e-partograph were appropriately being managed or referred in cent percentage of cases. The e-partograph is designed with the attached time stamp, and therefore, unlike like paper-partograph, could not be filled out by nurse- midwives after delivery has taken place. [30] Similar to our study findings, the e-partograph was recognized and recommended as an attractive and important tool for routine monitoring of labor in hospitals in other resource-poor settings [23, 30]

With the increased use of e-Partograph, this study has also observed the reduced rate of prolonged labour and related birth complications. While both paper- and electronic-partograph were effective in documenting the normal progress of labour in our study, the percentage of delivery outcomes occurring before reaching the alert line was higher for e-partograph (95%) than paper partograph (93%). Moreover, the labours monitoring through e-partograph, crossed the action line less frequently than paper partograph (2% Vs 4.5%) and no delivery cases crossed the action line in e-partograph group This finding is consistent with the other studies done in India among public health facilities [31–33]. As we know, the cervical graph under WHO partograph has specifically designed to capture the labour progression using action and the alert lines. The cervical dilation in centimeters per hours recorded during the active phase at two hours ineterval has been used to plot the cervical line. Two cervical lines, “Alert and Action line” are in built within the partograph to guide the expectant labour progression which were standardized by clinical trials findings. If any cervical plotting is on or left to alert line means progress of labour is good. If any plots are on the right side of alert line or the action line, meaning quick decision is needed for immediate termination of pregnancy. Using the online real time data source of the e-partograph platform, doctors were able to access the patient’s clinical condition from outside the labour room and could advise further treatment plans accordingly. Moreover, our study findings demonstrate that e-partograph could signal poor labor progression better (than paper partograph) that helped health care providers to initiate obstetric interventions on time such as artificial rupture of membranes and labour augmentation on time, could reduce prolonged labour and related complications. Our data confirms that the rate of prolonged labour was less with e-partograph than with the paper partograph (42% vs 30%). The effect of partograph use upon reduction of prolonged labour has been reported by other studies as well [31, 32]. Reduction of prolonged labour with use of both types of partograph might be due to training on partograph use for the delivery caregivers.

Whilst other authors have cautioned that the use of the partograph may increase the rate of obstetric interventions including C-sections [4, 5], our study documented that facility based C-section rates decreased by 6% in the Jessore district hospitals and by 11% in the Kushtia district hospitals during one-year study period. From our study finding we may hypothesize that monitoring labour through the e-partograph, backed up by management guidelines, improved compliance to the labour management protocol, contributed to the reduction in C-section rates. Similar findings have also been observed with the paperless partograph used in other country contexts. Deka G et al. reported that C-section rate was 10% with the WHO paper partograph while it reduced to 6% with the paperless partograph [34]. C-section deliveries, as a component of emergency obstetric care was designed to save maternal and fetal lives when the delivery course gets complicated and under certain clinical conditions. WHO recommends that population based C-section rate should be between10-15% [35]. However, an epidemic of C-section delivery is observed and in many LMICs the population based C-section rate has continue to rise far above the recommended 15% and that is a major public health concern in many countries [36]. The recent study from WHO has conferred that C-section rate above 19% had negative impact on maternal and neonatal mortality [36]. However, the facility based C-section are found to vary, based on the obstetric risk of women attending there, type of hospital (secondary vs. tertiary; and public vs. private), patient preferences and financial incentives for providing elective C-sections. While partograph cannot address the ‘financial’ and ‘preference’ drivers of elective C-sections, our study findings confirm that it is an effective tool to monitor pregnant women effectively, and ensure that those who need a C-section are correctly identified in a timely fashion. Thus the strict monitoring of the delivering women can only guarantee the safest use of C-section where partograph can play a significant role [37].

Bangladesh could make remarkable progress in reducing the MMR and neonatal mortality rates. The child health target set by millennium development goal (MDG) 4 was achieved before the target year 2015 [38]. Although the maternal mortality ratio (MMR) has decreased 40% in the last one decade, we failed to achieve MDG target on maternal health[38]. The MMR has been stalled since 2010 at 194 per 100,000 live-births and haemorrhage and eclampsia remain the major killers of women in Bangladesh [39]. Though the death related to prolonged labour has decreased [39] the current population based C-section rate (31%) is much higher than the WHO recommended 15%. In this context experts opined that ensuring quality delivery care can only improve the MMR status in Bangladesh. Our study suggests that routine use of partograph during delivery in hospital settings can play significant role in identification of delivery complications and their timely management.

Electronic versions of the partograph have recently been recommended as an effective tool, in low resource settings where human resource shortages are a concern, to offer one to one labour monitoring services at the health facility [23]. Bangladesh is in an advantageous position to adopt digital technology as online data entry, analysis and feedback and use has already been practiced through introduction of DHIS-2 software developed by Oslo University since 2009[40]. All public sector hospitals have internet facilities with adequate supply computers, laptops, and tablets to collect aggregate and individual level data which is sent regularly to the central server located at the Management Information System (MIS) unit in the office of the Director General of Health Services (DGHS). A statistician is there in each district and sub-district who are already trained to provide onsite technical support. All in-patients’ case records and clinical diagnoses are also being uploaded to the server using the event capture form. Under this initiative, a number nurse-midwives and doctors from district and sub-district hospitals have also been trained on data entry, analysis and feedback along with patient record management [40, 41]. Thus, the proposed nationwide implementation of e-partograph will not add any extra burden upon the health system.

### Strengths and limitations

The crossover design is the strength of this study which helps to mitigate potential health system biases. Care was taken to include equal numbers of deliveries from morning, evening, and night shifts, but this was not always possible due to the nature of delivery and available resources for this study.

## Conclusions

This study demonstrates that the partograph user rate significantly improved with the introduction of e-partograph. Implementing the e-partograph is feasible and effective in the Bangladeshi context where e-health and m-health interventions are already in place. We recommend inclusion of e-partograph in the labour management guidelines, and as a job aid for delivery attendants to support timely decision making for emergency obstetric interventions. Scaling e-partograph has the potential to improve the quality of maternal and neonatal health care services in Bangladesh, and could reduce the prevalence of un-necessary C-sections, birth asphyxia and prolonged labour along with related complications.

## Supporting information

S1 Table Institutional review board approved research protocol. (PDF)

S2 Table TREND statement checklist. (PDF)
