# Supplementary material for: Feasibility and effectiveness of electronic vs. paper partograph on improving birth outcomes: A prospective crossover study design
Source: PLoS One. 2019 Oct 7;14(10):e0222314. doi: 10.1371/journal.pone.0222314 (PMC6779270; doi:10.1371/journal.pone.0222314)
Supplement: S1 Table — (PDF) [file pone.0222314.s001.pdf]

| icddr,b | RRC APPLICATION FORM |
|---|---|

**RESEARCH PROTOCOL**
**Number: PR-14011**
**Version No. 3.00**
**Version date: 5.01.2015**

**FOR OFFICE USE ONLY**

| | | | |
|---|---|---|---|
| RRC Approval: | ☒ Yes | ☐ No | Date:19.11.2014 |
| ERC Approval: | ☐ Yes | ☐ No | Date: |
| AEEC Approval: | ☐ Yes | ☐ No | Date: |
| External IRB Approval | ☐ Yes | ☐ No | Date: |
| Name of External IRB: ____________ | | | |

**Protocol Title:* (**maximum 250 characters including space**)** Electronic partograph: A way of improving partograph use during labour monitoring process in selected district hospitals in Bangladesh

**Short Title: (**maximum **100** characters including space) Introducing an electronic partograph in district hospitals in Bangladesh

**Key Words:*** E-partograph, maternal health, labour, district hospital, Bangladesh

**Name of the Centre Hosting the Protocol:***

☐ Child and Adolescent Health
☐ Chronic Diseases
☐ Communicable Diseases
☐ Equity and Health Systems
☐ Food and Waterborne Diseases
☐ HIV/ AIDS
☐ Nutrition and Food Security
☐ Population, Urbanisation and Climate Change
☒ Reproductive Health
☐ Vaccine Sciences
☐ Other (specify)

**Has the Protocol been Derived from an Activity:*** ☒ No ☐ Yes (please provide following information):

Activity No. :
Activity Title:
PI:
Grant No.: Budget Code: Start Date: End Date:

**SP2020 Research Priority Area:* (**check all that apply**)**

☐ Healthy Life Course
☒ Mitigating Risk and Vulnerability
☐ Combating Priority Diseases
☐ Equitable Health Systems

**Research Phase (4 Ds):* (**check all that apply**)**

☒ Discovery
☒ Development
☐ Delivery
☐ Evaluation of Delivery

**Anticipated Impact of Research:* (**check all that apply**)**

☒ Knowledge Production
☒ Capacity Building
☐ Informing Policy
☒ Health and Health Sector Benefits
☐ Economic Benefits

**Which of the Millennium Development Goal(s) this Protocol Relates to:* (**check all that apply**)**

☐ 1. Eradicate Extreme Poverty and Hunger
☐ 3. Promote Gender Equality and Empower Women
☒ 5. Improve Maternal Health
☐ 7. Ensure Environmental Sustainability
☐ 2. Achieve Universal Primary Education
☒ 4. Reduce Child Mortality
☐ 6. Combat HIV/AIDS, Malaria and Other Diseases
☐ 8. Develop a Global Partnership for Development

**Which of the HPNSDP Programme Priorities this Protocol Relates to**:* (check all that apply)

☒ Scaling up Services to Achieve MDGs 1, 4, 5 and 6
☐ Addressing Population Growth with FP Services and Multi-sector Interventions
☐ Mainstreaming Nutrition in all Service Delivery Points through DGHS and DGFP
☐ Expanding Access to Priority Communicable and Non Communicable Diseases Services
☐ Revitalizing Community Clinic based Services under Upazila Health System
☐ Strengthen Health System & Governance, Sustainable M & E System and Health Information System
☐ Improving Health Equity for the Poor and Marginalized Population

| | |
|---|---|
| **Does this Protocol use the Gender Framework:***<br>(Please visit: http://www.icddrb.net.bd/jahia/Jahia/pid/684 for Gender Alanysis Tool with instructions) | ☒ Yes (please complete Gender Analysis Tool)<br>☐ No |
| If 'no' is the response, its reason(s) in brief: | |
| **Will this Research Specifically Benefit the Disadvantaged (**economically, socially and/or otherwise**):** | ☒ Yes<br>☐ No |
| **Does this Protocol use Behaviour Change Communication:** | ☐ Yes<br>☒ No |
| **Principal Investigator (Should be icddr,b staff):*** Sex ☐ Female ☒ Male<br>**Dr. Aminur Rahman**<br>(Position, phone no, extension no, cell, and email address **):**<br>Assistant Scientist,9840523-32, ex: 2200, 01713257399, draminur@icddrb.org | Primary Centre of PICentre for Reproductive Health (CRH) |
| **Co-Principal Investigator(s) Internal**: Sex ☐ Female ☒ Male<br>**Dr. Iqbal Anwar**<br>(Position, phone no, extension no, cell, and email address **):**<br>Associate Scientist, 9840523-32, ex: 2200, iqbal@icddrb.org | Primary Centre of Co-PI<br>Centre for Reproductive Health (CRH) |
| **Co-Investigator(s) - Internal:** Sex ☒ Female ☐ Male<br>**Sadika Akhter**<br>Deputy Pragramme Coordinator,9840523-32, ex: 2201, sadika@icddrb.org<br>[if more than one, please copy and paste this row for additional Co-Is | Primary Centre of Co-I<br>Centre for Reproductive Health (CRH) |
| **Co-Investigator(s) - Internal:** Sex ☐ Female ☒ Male<br>**Dr. Dewan Md. Emdadul Haque**<br>(Position, phone no, extension no, cell, and email address **):**<br>Associate Scientist & Project Coordinator, emdad@icddrb.org<br>[if more than one, please copy and paste this row for additional Co-Is | Primary Centre of Co-ICentre for Child and Adolescent Health (CCAH) |
| **Co-Investigator(s) - Internal:** Sex ☐ Female ☒ Male<br>**Monjur Rahman**<br>(Position, phone no, extension no, cell, and email address **):**<br>Senior Programmer, 01714080465, monjur@icddrb.org<br>[if more than one, please copy and paste this row for additional Co-Is | Primary Centre of Co-ICentre for Equity and Health Systems (CEHS) |
| **Co-Investigator(s) - Internal:** Sex ☐ Female ☒ Male<br>**Sumon Kumar Das**<br>(Position, phone no, extension no, cell, and email address **):**<br>Senior Medical Officer /Assistant Scientist, sumon@icddrb.org<br>[if more than one, please copy and paste this row for additional Co-Is | Primary Centre of Co-ICentre for Nutrition and Food Security (CNFS) |
| **Co-Investigator(s) – External:** Sex ☒ Female ☐ Male<br>**Address** (provide full official address, including land phone no(s), extension no. (if any), cell phone number, and email address):<br>Prof. Fatema Ashraf<br>Dept. of Obs and Gynae, ShSMC, 01739767778, fatema_ashraf@yahoo.com | |
| **Co-Investigator(s) – External:** Sex ☒ Female ☐ Male<br>**Address** (provide full official address, including land phone no(s), extension no. (if any), cell phone number, and email address):<br>Prof. Perveen fatima, Professor of Infertility, BSMMU. Email:pfatima@citech.net, drpfatima@gmail.com,<br>Mobile: +88-01819216927 | |
| **Student Investigator(s) - Internal:** Sex ☐ Female ☐ Male<br><br>(Position, phone no, extension no, cell, and email address **):** | Students Affiliation |
| **Student Investigator(s) - External:** Sex ☐ Female ☐ Male<br>Address (provide full official address, including land phone no(s), extension no. (if any), cell phone number, and email address): | |

**Collaborating Institute(s):** Please provide full official address

**Institution # 1**

| | |
|---|---|
| Country | Bangladesh |
| Contact person | Dr. SAJ Md Musa |
| Department<br>(including Division, Centre, Unit) | Director, Maternal, Neonatal, Child and Adolescent Health |
| Institution<br>(with official address) | Directorate General of Health Services (DGHS) |
| Directorate<br>(in case of GoB i.e. DGHS) | Directorate General of Health Services (DGHS) |
| Ministry (in case of GoB) | Ministry of Health and Family Welfare |

**Institution # 2**

| | |
|---|---|
| Country | |
| Contact person | |
| Department<br>(including Division, Centre, Unit) | |
| Institution<br>(with official address) | |
| Directorate<br>(in case of GoB i.e. DGHS) | |
| Ministry (in case of GoB) | |

**Institution # 3**

| | |
|---|---|
| Country | |
| Contact person | |
| Department<br>(including Division, Centre, Unit) | |
| Institution<br>(with official address) | |
| Directorate<br>(in case of GoB i.e. DGHS) | |
| Ministry (in case of GoB) | |

Note: If less than or more than three collaborating institutions, please delete or insert blocks as needed.

**Contribution by the Members of the Scientific Team:**

| Members' Name | Contribution | | | | | | | | |
|---|---|---|---|---|---|---|---|---|---|
| | Research idea/ concept | Study design | Protocol writing | Respond to external reviewers' comments | Defending at IRB | Developing data collection Tool(s) | Data Collection | Data analysis/ interpretation of results | Manuscript writing |
| Dr. Aminur Rahman | ☒ | ☒ | ☒ | ☒ | ☒ | ☒ | ☐ | ☒ | ☒ |
| Dr. Iqbal Anwar | ☐ | ☒ | ☒ | ☒ | ☒ | ☒ | ☐ | ☒ | ☒ |
| Sadika Akhter | ☐ | ☒ | ☒ | ☒ | ☒ | ☒ | ☐ | ☒ | ☒ |
| Dr. Dewan Md. Emdadul Hoque | ☐ | ☒ | ☐ | ☐ | ☐ | ☒ | ☐ | ☒ | ☒ |
| Prof Fatema Ashraf | ☐ | ☐ | ☐ | ☐ | ☐ | ☒ | ☒ | ☐ | ☒ |
| Monjur Rahman | ☐ | ☒ | ☒ | ☐ | ☒ | ☒ | ☒ | ☐ | ☐ |
| Prof Perveen Fatima | ☐ | ☒ | ☒ | ☐ | ☐ | ☒ | ☒ | ☐ | ☒ |
| Dr. Sumon Kumar Das | ☐ | ☒ | ☒ | ☒ | ☐ | ☒ | ☐ | ☒ | ☒ |
| | ☐ | ☐ | ☐ | ☐ | ☐ | ☐ | ☐ | ☐ | ☐ |
| | ☐ | ☐ | ☐ | ☐ | ☐ | ☐ | ☐ | ☐ | ☐ |
| | ☐ | ☐ | ☐ | ☐ | ☐ | ☐ | ☐ | ☐ | ☐ |
| | ☐ | ☐ | ☐ | ☐ | ☐ | ☐ | ☐ | ☐ | ☐ |
| | ☐ | ☐ | ☐ | ☐ | ☐ | ☐ | ☐ | ☐ | ☐ |
| | ☐ | ☐ | ☐ | ☐ | ☐ | ☐ | ☐ | ☐ | ☐ |

**Study Population: Sex, Age, Special Group and Ethnicity**

**Research Subject:**
☒ Human
☐ Animal
☐ Microorganism
☐ Other (specify): ______________

**Sex:**
☒ Male
☒ Female
☐ Transgender

**Age:**
☐ 0 – 4 years
☐ 5 – 10 years
☒ 11 – 17 years
☒ 18 – 64 years
☐ 65 +

**Special Group:**
☒ Pregnant Women
☐ Fetuses
☐ Prisoners
☐ Destitutes
☒ Service Providers
☐ Cognitively Impaired
☐ CSW
☐ Expatriates
☐ Immigrants
☐ Refugee
☐ Others (specify): _________________

**Ethnicity:**
☒ No ethnic selection (Bangladeshi)
☐ Bangalee
☐ Tribal group
☐ Other (specify): _________________

**NOTE:** It is icddr.b's policy to include men, women, children and transgender in its research projects involving participation of humans, unless there is strong justification(s) for their exclusion.

**Consent Process: (**Check all that apply**)**

☒ Written
☐ Oral
☐ Audio
☐ Video
☐ None

**Language:**

☒ Bangla
☐ English
☐ Other (specify: ________________

**Project/Study Site: (**Check all that apply**)**

- ☐ Chakaria
- ☐ Bandarban
- ☐ Dhaka Hospital
- ☐ Kamalapur Field Site/HDSS
- ☐ Mirpur (Dhaka)
- ☐ Matlab DSS Area
- ☐ Matlab non-DSS Area
- ☐ Matlab Hospital
- ☐ Mirzapur
- ☐ Bianibazar (Sylhet)
- ☐ Kanaighat (Sylhet)
- ☐ Jakigonj (Sylhet)
- ☐ Other community in Dhaka
  Name: Two district hospitals
- ☒ Other sites in Bangladesh
  Name: District Hospitals
- ☐ Multi-national Study
  Name of the country______________________

**Project/Study Type:** (Check all that apply)

- ☐ Case Control Study
- ☐ Clinical Trial (Hospital/Clinic/Field)*
- ☐ Community-based Trial/Intervention
- ☐ Cross Sectional Survey
- ☐ Family Follow-up Study
- ☒ Longitudinal Study (cohort or follow-up)
- ☐ Meta-analysis
- ☐ Programme Evaluation
- ☐ Programme (Umbrella Project)
- ☐ Prophylactic Trial
- ☐ Record Review
- ☐ Secondary Data Analysis
  Protocol No. of Data Source: _____________
- ☐ Surveillance/Monitoring
- ☐ Systematic Review
- ☐ Other (specify): _________________________

***Note**: International Committee of Medical Journal Editors (ICMJE) defines Clinical Trial as "*Any research project that prospectively assigns human participants to intervention and comparison groups to study the cause-and-effect relationship between a medical intervention and a health outcome*".

PI of the RRC- and ERC-approved Clinical Trials should provide necessary information to IRB Secretariat (Research Administration) for registration and uploading into relevant websites (usually at the https://register.clinicaltrials.gov/). They should also provide relevant information to the IRB Secretariat in the event of amendment/modification after their approval by RRC and ERC.

**Biological Specimen:**

| | |
|---|---|
| a) Will the biological specimen be stored for future use? | ☐ Yes ☐ No ☒ Not applicable |
| b) If the response is 'yes', how long the specimens will be preserved? | __________ years |
| c) What types of tests will be carried out with the preserved specimens? | |
| d) Will the consent be obtained from the study participants for use of the preserved specimen for other initiative(s) unrelated to this study, without their re-consent? | ☐ Yes ☐ No ☒ Not applicable |
| e) Will the specimens be shipped to other country/ countries? If yes, name of institution(s) and country/ countries? | ☐ Yes ☐ No ☒ Not applicable |
| f) If shipped to another country, will the surplus/unused specimen be returned to icddr,b? If the response is 'no', then the surplus/unused specimen must be destroyed. | ☐ Yes ☐ No ☒ Not applicable |
| g) Who will be the custodian of the specimen at icddr,b? | |
| h) Who will be the custodian of the specimen when shipped outside Bangladesh? | |
| i) Who will be the owner(s) of the specimens? | |
| j) Has a MoU been signed with regards to collection, storage, use and ownership of specimen? If the response is 'yes', please attach a copy of the MoU.. If the response is 'no', appropriate justification should be provided for not signing a MoU. | ☐ Yes ☐ No ☒ Not applicable |

**Proposed Sample Size:**
Sub-group (Name of subgroup e.g. Men, Women) and Number

| Name | Number | Name | Number |
|---|---|---|---|
| (1) Deliveries that will be conducted in Kushtia DH | 506 | (3) | |
| (2) Deliveries that will be conducted in Jessore DH | 506 | (4) | |
| | | **Total sample size** | 1012 |

**Determination of Risk: Does the Research Involve** (Check all that apply)

- ☐ Human exposure to radioactive agents?
- ☐ Foetal tissue or abortus?
- ☐ Investigational new device?
  Specify: ___________________
- ☐ Existing data available from Co-investigator?
- ☐ Human exposure to infectious agents?
- ☐ Investigational new drug?
- ☐ Existing data available via public archives/sources?
- ☐ Pathological or diagnostic clinical specimen only?
- ☒ Observation of public behaviour?
- ☐ New treatment regime?

| | Yes | No |
|---|---|---|
| Will the information be recorded in such a manner that study participants can be identified from the information directly or through identifiers linked to the study participants? | ☐ | ☒ |
| Does the research deal with sensitive aspects of the study participants' sexual behaviour, alcohol use or illegal conduct such as drug use? | ☐ | ☒ |
| **Could information on study participants, if available to people outside of the research team:** | | |
| a) Place them at risk of criminal or civil liability? | ☐ | ☒ |
| b) Damage their financial standing, reputation or employability, or social rejection, or lead to stigma, divorce etc.? | ☐ | ☒ |

**Do you consider this research:** (check one)

| | | |
|---|---|---|
| ☐ Greater than minimal risk | ☒ No more than minimal risk | ☐ Only part of the diagnostic test |

**Note: Minimal Risk:** The probability and the magnitude of the anticipated harm or discomfort to participants is not greater than those ordinarily encountered in daily life or during the performance of routine physical, psychological examinations or tests, e.g. the risk of drawing a small amount of blood from a healthy individual for research purposes is no greater than when the same is performed for routine management of patients.

| **Risk Group of Infectious Agent and Use of Recombinant DNA** | | | | |
|---|---|---|---|---|
| a) Will specimens containing infectious agent be collected? | ☐ Yes | ☐ No | ☒ Not applicable | |
| b) Will the study involve amplification by culture of infectious agents? | ☐ Yes | ☐ No | ☒ Not applicable | |
| c) If response to questions (a) and/or (b) is 'yes', to which Risk Group (RG) does the agent(s) belong? (Please visit http://www.icddrb.net.bd/jahia/Jahia/pid/684 to review list of microorganism by Risk Group) | ☐ RG1 | ☐ RG2 | ☐ RG3 | ☐ RG4 |
| d) Does the study involve experiments with recombinant DNA? | ☐ Yes | ☐ No | ☒ Not applicable | |

**Does the study involve any biohazards materials/agents or microorganisms of risk group 2, 3, or 4 (GR2, GR-3 or GR4)?**

☐ Yes ☒ No

[If the response is 'yes'] I, (print name of the PI) affirm that we will use the standard icddr,b laboratory procedures for biosafety of the hazardous materials/agents or microorganisms in the conduction of the study.

________________________________ 29.01.2014

**Signature of the Principal Investigator** **Date**

**Dissemination Plan:** [please explicitly describe the plans for dissemination, including how the research findings would be shared with stakeholders, identifying them if known, and the mechanism to be used; anticipated type of publication (working papers, internal (institutional) publication, international publications, international conferences/seminars/workshops/ agencies. [Check all that are applicable]

| Dissemination type | Response | | Description (if the response is a yes) |
|---|---|---|---|
| Seminar for icddr,b scientists/ staff | ☒ No | ☐ Yes | |
| Internal publication | ☒ No | ☐ Yes | |
| Working paper | ☒ No | ☐ Yes | |
| Sharing with GoB (e.g. DGHS/ Ministry, others) | ☐ No | ☒ Yes | A dissemination session will be organized at DG health with participation from GoB personnel, NGOs & professional bodies |
| Sharing with national NGOs | ☐ No | ☒ Yes | DO |
| Presentation at national workshop/ seminar | ☐ No | ☒ Yes | Availability of extra fund presentation will be delivered at international workshop/seminar |
| Presentation at international workshop/ conference | ☐ No | ☒ Yes | Abstract will be submitted in international conference to present the study |
| Peer-reviewed publication | ☐ No | ☒ Yes | A manuscript will be developed for peer reviewed publication |
| Sharing with international agencies | ☐ No | ☒ Yes | They will be invited during dissemination session at DGHS |
| Sharing with donors | ☐ No | ☒ Yes | As per mentioned in RFP |
| Policy brief | ☒ No | ☐ Yes | |
| Other | | | |
| Other | | | |

**Funding:**

| | | |
|---|---|---|
| Is the protocol fully funded? | ☒ Yes | ☐ No |
| If the answer is yes, please provide sponsor(s)'s name | 1. DFID MNH innovation & Quality of Care | |
| | 2. | |
| Is the protocol partially funded? | ☐ Yes | ☒ No |
| If the answer is yes, please provide sponsor(s)'s name | 1. | |
| | 2. | |

**If fund has not been identified:**

| | | |
|---|---|---|
| Is the proposal being submitted for funding? | ☐ Yes | ☐ No |
| If yes, name of the funding agency | 1. | |
| | 2. | |

**Conflict of interest:**
Do any of the participating investigators and/or member(s) of their immediate families have an equity relationship (e.g. stockholder) with the sponsor of the project or manufacturer and/or owner of the test product or device to be studied or serve as a consultant to any of the above?

☒ No | ☐ Yes (please submit a written statement of disclosure to the Executive Director, icddr,b)

**Proposed Budget:**

**Dates of Proposed Period of Support**

(Day, Month, Year - DD/MM/YY)

Beginning Date : 01.04.2014

End Date : 31.10.2015

**Cost Required for the Budget Period ($)**

| Years | Direct Cost | Indirect Cost | Total Cost |
|---|---|---|---|
| **Year-1** | 70153 | 7015 | 77168 |
| **Year-2** | 17,484 | 1748 | 19232 |
| **Year-3** | | | 0 |
| **Year-4** | | | 0 |
| **Year-5** | | | 0 |
| **Total** | 87637 | 8763 | 96400 |

**Certification by the Principal Investigator:**

I certify that the statements herein are true, complete and accurate to the best of my knowledge. I am aware that any false, fictitious, or fraudulent statements or claims may subject me to criminal, civil, or administrative penalties. I agree to accept the responsibility for the scientific conduct of the project and to provide the required progress reports including updating protocol information in the NAVISION if a grant is awarded as a result of this application.

I also certify that I have read icddr,b Data Policies and understand the PIs' responsibilities related to archival and sharing of research data, and will remain fully compliant to the Policies. (Note: The Data Policies can be found here: http://www.icddrb.org/who-we-are/data-policies)

_____________ 29.01.2014

**Signature of PI** **Date**

**Approval of the Project by the Centre Director of the Applicant:**

The above-mentioned project has been discussed and reviewed at the Centre level.

| Centre for Reproductive Health | ____________ | 29.01.2014 |
|---|---|---|
| Name of the Centre Director | Signature | Date of Approval |

# Table of Contents


RRC APPLICATION FORM.....1
Project Summary.....10
Hypothesis to be tested:.....11
Specific Objectives:.....11
Background of the Project including Preliminary Observations:.....11
Research Design and Methods.....15
Sample Size Calculation and Outcome (Primary and Secondary) Variable(s).....23
Data Analysis.....24
Data Safety Monitoring Plan (DSMP).....24
Ethical Assurance for Protection of Human rights.....25
Use of Animals.....25
Collaborative Arrangements.....25
Facilities Available.....25
Literature Cited.....25
Budget.....28
Other Support.....29
Biography of the Investigators.....30
Format for Consent Form.....43
Check-List.....71


☒ Check here if appendix is included

## Project Summary

[The summary, within a word limit of 300, should be stand alone and be fully understandable.]


Principal Investigator: Dr. Aminur Rahman

Research Protocol Title: Electronic partograph: A way of improving partograph use during labour monitoring process in selected district hospitals in Bangladesh

Proposed start date: 01.07.2014 Estimated end date: 31.01.2016

**Background** (brief):

a. Burden: Abnormal prolonged labour and its effects are important contributors to maternal and perinatal mortality and morbidity worldwide. Although the partograph has been shown to be an efficacious tool for monitoring labor and identifying women in need of an comprehensive emergency obstetric intervention, it's appropriate use is questionable throughout the world. Evidence suggests that very few service provider use partograph despite recognizing its usefulness.
b. Knowledge gap: In Bangladesh partograph is hardly used by the concern person though its importance and positive outcome is acknowledged at the national level. To introduce a digital partograph Johns Hopkins Program for International Education in Gynecology and Obstetrics (Jhpiego) developed an ePartogram device and WHO created partograph e-Learning tool. Jhpiego is currently testing three ePartogram implementations. However, till today, no data has been collected or analysed for any of these ePartogram models. The WHO e-Learning tool has been distributed to facilities like Kenyatta National Hospital via CD-ROM. In Kenya, a study also being conducted using a digital partograph (partopen) and results are yet to come.
c. Relevance: The use of the paper partograph is found to be complex and too much time-consuming for effective use in low-resource settings where there has been inadequate health care staffing. These challenges highlight a need for strengthening providers' skills and/or developing new technologies that are suitable for low-resource countries and promote consistent, correct use of the partograph as well as, a labour monitoring system for the peripheral level health facilities. The benefits of this device are: ease of use, minimal training requirements, improved data quality and capture, and seamless integration into current local practice.

**Objectives**: The current study aims-

1. To explore the feasibility, acceptability of digital partograph use among health service providers in selected district hospitals in Bangladesh;
2. To compare the user rate between paper and digital partograph during labour monitoring process in district hospitals in Bangladesh;
3. To compare the outcome of paper and digital partograph use in respect to the birth asphyxia and prolonged labour rate after introducing digital partograph in selected district hospitals in Bangladesh;
4. To determine the barriers and facilitating factors of introducing electronic partograph in district hospital in Bangladesh.

**Methods**: A mixed-method prospective follow-up study with crossover design will be conducted in two DHs. One DH will serve as intervention (electronic partograph) hospital and another as control hospital (paper-based partograph). After applying the crossover design this allocation will be reversed. The total study will be completed within 18 months period. Nurse-midwives posted in obstetric wards will be the study participants. The sample size in each DH will be 506 mothers who will deliver their baby during the study period.

**Outcome measures/variables**:

- Feasibility and acceptability of digital partograph use will be assessed among health service providers in selected DH in Bangladesh;
- The user rate of digital partograph will be estimated during labour monitoring process in DHs in Bangladesh;
- The birth asphyxia and prolonged labour rate will be estimated after introducing digital partograph in selected DHs in Bangladesh;
- The barriers and facilitating factors of introducing electronic partograph will be determined in DHs in Bangladesh.

# Description of the Research Project

## Hypothesis to be tested:

In a hypothesis testing research proposal, briefly mention the hypothesis to be tested and provide the scientific basis of the hypothesis, critically examining the observations leading to the formulation of the hypothesis.

Does this research proposal involve testing of hypothesis: ☒ No ☐ Yes (describe below)

## Specific Objectives:

Describe the specific objectives of the proposed study. State the specific parameters, gender aspects, biological functions, rates, and processes that will be assessed by specific methods.

1. To explore the feasibility and acceptability of digital partograph use among health service providers in selected district hospitals in Bangladesh;
2. To compare the user rate between paper and digital partograph during labour monitoring process in the study hospitals in Bangladesh;
3. To compare the outcome of paper and digital partograph use in respect to the birth asphyxia and prolonged labour rate after introducing digital partograph in selected district hospitals in Bangladesh;
4. To determine the barriers and facilitating factors of introducing electronic partograph in district hospital in Bangladesh.

## Background of the Project including Preliminary Observations:

Provide scientific validity of the hypothesis based on background information of the proposed study and discuss previous works on the research topic, including information on sex, gender and diversity (ethnicity, SES) by citing specific references. Critically analyze available knowledge and discuss the questions and gaps in the knowledge that need to be filled to achieve the proposed aims. If there is no sufficient information on the subject, indicate the need to develop new knowledge.

Annually, an estimated 287,000 maternal and 3.1 million neonatal deaths occur, 99% of which are in developing countries [1, 2]. A large proportion of these deaths follow prolonged labour, due mainly to cephalopelvic disproportion, a condition in which the newborn is too large to fit through the mother's pelvis, resulting in obstructed labour, maternal dehydration, ruptured uterus, obstructed fistula and postpartum hemorrhage (PPH). For the newborn, prolonged obstructed labour may cause asphyxia, brain damage, infection and death during the neonatal period or later. Obstructed labour with or without rupturing the uterus ranks among the five major causes of maternal deaths in almost every developing country, although its relative importance varies from region to region. Nevertheless, it can be said with certainty that abnormal prolonged labour and its effects are important contributors of maternal and perinatal mortality and morbidity worldwide [3].
There are many constraints which can lead to non-availability or non-utilization of basic obstetric care needed to manage obstructed labour satisfactorily. Early detection of abnormal progress and prevention of prolonged labour would help to reduce maternal and perinatal mortality. One such tool that has been shown to be an effective for monitoring labour and identifying women in need of an obstetric intervention is the partograph [3].

### The partograph: A labour monitoring tool

The partograph, a framework for assessing maternal and foetal condition and labour progression, dates back to the 1950s. The concept of the partograph and its labor curves was first conceived by E.A. Friedman, an obstetrician, who used it to monitor cervical dilation [4]. In 1972, R.H. Philpott improved upon Friedman's partograph by designing alert lines and action lines to help capture prolonged labour, which allowed the partograph to serve as a practical tool for recording all intrapartum observations in addition to cervical dilation= [5]. In 1988, Safe Motherhood Initiative launched the use of the partograph as an international standard for monitoring and preventing prolonged labour. In 1994, WHO extensively tested its efficacy and established the scientific basis and rationale for its use in prevention of prolonged labour [6, 7]. It was found that its use reduces the incidence of prolonged and obstructed labour and can also detect foetal heart abnormalities that

may result in intrapartum foetal hypoxia. Subsequently, in 1994, WHO declared the universal application of the partograph in all settings [7]. (Annex: 9).
Several maternal conditions are monitored to assess the well being of the mother. For instance, maternal condition is observed by checking blood pressure to detect pre-eclampsia and eclampsia; pulse rate is observed to detect dehydration, blood loss or sepsis during labour; temperature is checked to identify raised temperature which indicates sepsis; and urine output is checked to exclude proteinuria and dehydration and to keep the bladder empty as a full urinary bladder obstructs foetal head descent. If a mother's well-being is compromised, the foetal condition is also compromised. As a result, certain conditions may not allow both lives to be saved. [8].

Likewise, the foetal conditions can be monitored to assess the well being of the unborn child. Foetal Heart Rate (FHR) monitoring is assumed to identify a baby's risk of running short of oxygen (hypoxia). State of membranes show the risk of baby and mother to ascending infections if ruptured for long. The colour of liquor can tell whether the foetal life is compromised or not. The health worker would anticipate vaginal delivery if there is no excessive moulding and caput. Labour progress is captured by monitoring cervical dilatation, which indicates whether labour is precipitated, normal or prolonged. Precipitated and prolonged labours are potential risks of post-partum haemorrhage (PPH) [8]. Meaningful interpretation of the cervical dilatation is aided by alert and actions lines on the graph. Alert lines are graphical lines drawn from 4cm to 10cm dilatation. The role of the alert line is to separate normal labour from abnormal labour. Crossing the alert line is associated with foetal distress which increases the need for resuscitation of the baby [9]. Action lines are graphical lines drawn four hours to the right of the alert line. The action line represents slow labour progress. Slow, prolonged labours are a potential source of maternal dehydration, exhaustion, uterine rupture and maternal and foetal sepsis [8, 10]. Contractions which are efficient, adequate and effective predict a normal progression of labour. Consistent and regular monitoring of contractions can show whether progression of labour is normal or not. If not, interventions can be instituted through augmentation of labour. Descent shows compatibility of foetal head and pelvis and failure of presenting part to descend in presence of strong contractions indicates cephalopelvic disproportion (CPD) which is the common cause of obstructed labour [5, 8]. Consistent and regular monitoring of descent can guide the health worker through the progress of labour and indicate whether it is satisfactory in anticipation of the time of delivery.

**Evolution of Partographs from the World Health Organization:**
The WHO has further modified and designed three partographs. 1) Composite partograph: includes a latent phase of 8 hours and an active phase that starts at a cervical dilation of 3 cm. 2) Modified partograph (adapted in 2000, for use in hospitals): excludes the latent phase and the active phase starts at a cervical dilation of 4 cm. 3) Simplified partograph (further adapted in 2000 for use by skilled attendants in health centers): excludes the latent phase and descent of the presenting part; the active phase starts at a cervical dilation of 4 cm. This comes color-coded.[11, 12]

**Barriers and challenges for using partograph:**
The partograph is regarded as a valuable tool, which may prevent prolonged labour and promote timely decisions during labour [13, 14], and is recommended for use in both peripheral and central labour units. However, it's appropriate use is questionable throughout the world. There are worries that the use of the partograph may increase the rate of obstetric interventions [4, 5]. However, an opposite result has been documented where successful use of the partograph is reported from a study conducted in India [15]. A survey from a labour unit of Zambia further reports that partographs are rarely used and is frequently missing in women's childbirth records [16]. A study in Nigeria revealed that very few obstetricians and midwives used partographs despite recognizing its usefulness [16]. Several factors have been documented for not using the partograph during labour

monitoring process, such as: poor knowledge of how to use partographs for labour management by midwives and other health workers, lack of supply of partographs, resistance to the use of the partograph, lack of a uniform protocol for the use of the partograph in tertiary level labour care and a lack of support from the health system [17, 18]. If use of the partograph is not properly introduced and supervised, midwives and other health workers might perceive this documentation as an administrative constrain [19].

**Proposed solution and potentiality to contribute in health system:**

Labor is a stressful time for a pregnant woman and her baby. While most women and babies tolerate labor well, some women will experience complications, and some fetuses will become distressed. The goal of monitoring the woman and fetus during labor is to ensure early identification and timely management of problem to prevent short- and long-term morbidity and mortality. Monitoring labor using a partograph allows health care providers to record, interpret, analyze, and use data to make decisions on labor management. Unfortunately, complete documentation on the partograph is extremely limited in low-resource countries including  Bangladesh [20]. The potential relationship between paper-based systems and digital tools is already proven, particularly mobile phone tools have been designed to simplify data collection [21, 22], improve community health worker performance and effectiveness [23-27].The benefits of this device are ease-of-use, minimal training requirements, improved data quality and capture, and seamless integration into current local practice. However, technologies are also used in health service delivery in Bangladesh. The service providers are becoming familiar in using technology in their service delivery.

It is perceived that the partograph is complex and too time consuming for effective use in low-resource countries that have inadequate health care staffing. These challenges highlight a need for strengthening providers' skills and/or developing new technologies that are suitable for low-resource countries and promoting consistent, correct use of the partograph as well as identification of labour monitoring systems that could be used at the peripheral level. This proposed study is aimed at improving quality of care and reducing mortality and morbidly during and around delivery and has potential to be linked with the current health system at public health facilities in Bangladesh

Prior research on improving the paper partograph form includes the ePartogram device developed by Johns Hopkins Program for International Education in Gynecology and Obstetrics (Jhpiego) [19], and the partograph e-Learning tool created by the WHO [9]. Jhpiego is currently testing three ePartogram implementations, which include an Android tablet application, a digital clipboard system, and a custom hardware solution, but at this time, no data has been collected or analysed for any of these models. The WHO e-Learning tool for partograph is distributed to facilities like Kenyatta National Hospital via CD-ROM. However, the CD-ROMs are not given to every student or directly incorporated into the nursing curriculum. Single copies of the tool are often passed from student to student throughout the academic year, placing the primary responsibility for learning the material upon the students themselves. In Kenya, an ongoing study is also being conducted using a digital partograph (partopen).

In Bangladesh although the use of partograph is recommended after each institutional delivery however its utilization is very poor. A study conducted in Obstetrics and Gynae department of Dhaka Medical College Hospital (DMCH)  has shown that using the partograph is highly effective in reducing complications from prolonged labour for the mother and for the newborn[28]. Another study conducted in the health facilities of Khulna and Sylhet divisions, showed that partograph user rate was only 5% to 33% [29]. However, a baseline survey among the nurse-midwives working in five types of maternity facilities from upazilla to tertiary level in Bangladesh revealed that  although more than two-thirds of the respondents (78.9 %) had the knowledge about what information that

needs to be recorded in a partograph, more than half of the respondents (51.4%) did not use the tool[30]. A recent needs assessment study conducted in 24 districts of Bangladesh also revealed that Partograph usage rate was only 3% among all institutional deliveries[31]. Also a multicenter retrospective record review in five low-income countries including Bangladesh revealed a disappointingly low user rate of partograph in Bangladesh which is only 1.4%[32]. However, to our knowledge, there is no study conducted using electronic partograph for labour monitoring in hospital settings in Bangladesh.

**Feasibility and acceptability:**

Feasibility concerns the ease of administration and processing of an instrument. These are important considerations for staff and researchers who collect and process the information produced by patient-reported instruments[33, 34].

Acceptability is the extent to which an instrument is acceptable to patients. Indicators of acceptability include administration time, response rates, and levels of missing data[35]. There are a number of factors that can influence acceptability including the mode of administration, questionnaire design, and the health status of respondents. The format of patient-reported instruments can also influence acceptability.

To capture the information of feasibility and acceptability the proposed study will stick with the dynamics of feasibility & acceptability those are mentioned in the above definitions and will develop the measuring tool accordingly.

**Future opportunities to use technology at district level in health sector in Bangladesh:**

Currently in Bangladesh, all district hospitals are involved in online data entry for maternal and child health care. They have internet facilities to connect with the central server which is placed at MIS of DGHS and collected data are sent regularly which are stored at the central server. A statistician is placed at district hospital and officially assigned for this task. The government has planned to introduce an online data entry system for indoor patient care at district hospitals. The nurses and doctors will get training for routing patient management records. Thus, there is potential to introduce electronic devices for health care at district hospitals in Bangladesh.

This operational research protocol has planned to introduce digital partograph to increase the user rate during labour monitoring process and explore the challenges of its introduction for uses by nurses and midwives in selected district hospitals. Moreover, the proposed study will be able to address the reported barriers through:

1. Poor knowledge on how to use partograph: The poor knowledge on partograph will be mitigated through proposed training & refreshers on partograph for the nurses and midwives under the current study. Moreover, by forming the local expert group from doctors and consultants will be able to provide the technical input and supportive supervision to the nurses and midwives.
2. Lack of supply: During the project period the study will ensure the supplies of paper partograph and will make liaison with the reproductive health programme manager at directorate office for the continuous supply. But introducing digital partograph might not need the paper partograph.
3. Lack of uniform protocol: The proposed study will ensure the availability of the standard protocol as the study aims to develop a standard user manual for partograph use following WHO and OGSB guideline.
4. Lack of supervision from the system: The electronic partograph will allow the authority to make an instant online quarry about the use of partograph for conducted deliveries.

5. Resistance to the use of the partograph: The proposed study is aimed to connect the user of partograph with the professionals and administrators who have potential to dilute the resistance not to use this tool during the labor monitoring process.

## Research Design and Methods

Describe the research design and methods and procedures to be used in achieving the specific aims of the research project. If applicable, mention the type of personal protective equipment (PPE), use of aerosol confinement, and the need for the use BSL2 or BSL3 laboratory for different part of the intended research in the methods.. Define the study population with inclusion and exclusion criteria, the sampling design, list the important outcome and exposure variables, describe the data collection methods/tools, and include any follow-up plans if applicable. Justify the scientific validity of the methodological approach (biomedical, social, gender, or environmental).

Also, discuss the limitations and difficulties of the proposed procedures and sufficiently justify the use of them.

**Study design, site and duration:**

It will be a mixed-method prospective follow-up with crossover study design [36, 37]. Two district hospitals have been selected after discussion with the Line Director, MNC&AH, DGHS. We have purposively chosen Kushtia District Hospital for electronic partograph and Jessore District hospital for paper based partograph to initiate the study. In Bangladesh, nurse-midwives conduct most of the facility deliveries under supervision of a qualified obstetrician and/or trained medical officers. So nurse-midwives posted in obstetric wards will be the study participant for the study who will observe the calculated deliveries during the study period. There are available district hospitals where on an average more than 1000 normal deliveries take place every year. The selected facilities would be similar in terms of infrastructure, human resources and service delivery scenario. The choices have been considered that these two hospitals are nearly similar in several characteristics and they are equally comparable. Both the hospitals have 250 beds and selected for medical colleges for teaching. The following table provides much detail information on this for the year 2012 (Table:2)

The proposed study sites are mostly similar in their performances except few, like; # of c-section, stillbirth rate. To minimize this non similarity the proposed study will follow a crossover study design. After enrolling half of the calculated sample; there will be a pause period and then crossover design will be introduced where the two sites will follow deliveries through the paper and electronic partograph just reverse of the initial plan. During the pause period training on paper and electronic partograph will be provided to the respective hospital to collect the information from the remaining sample (Table: 1).

Fig 1. Modality of study:

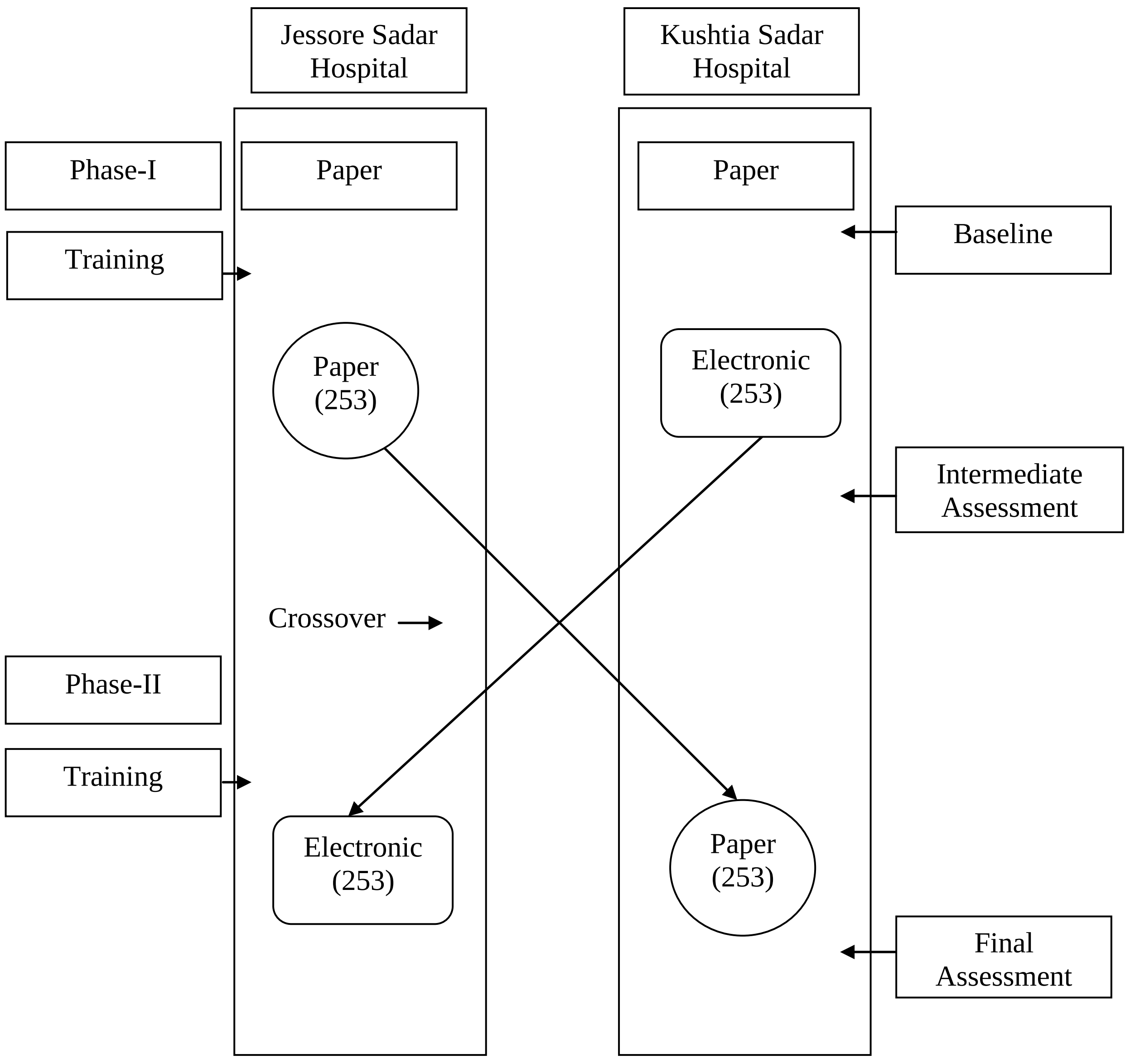

Table: 1 Comparative pregnancy related information both for Kushtia & Jessore sadar Hospital

| Name of hospital | Admitted patients in obs ward | Type of delivery | | | Outcome of delivery | | # of beds in obs ward | # nurses posted for Obs ward | # of doctors posted at Obs ward | Year of established |
|---|---|---|---|---|---|---|---|---|---|---|
| | | Normal delivery | Caesarean section | Total delivery | L ive birth | Stillbirth | | | | |
| Kushtia Sadar Hospital | 5,921 | 1,728 | 915 | 2,677 | 2,541 | 165 | 23 | 9 | 6 | 1963 |
| Jessore General Hospital | 4,107 | 1,327 | 1,498 | 2,852 | 2,755 | 118 | 20 | 11 | 9 | 1983 |

The selection procedure was consulted with Director MNC&AH of DGHS. The total study will be completed within 18 months period.

**Study instruments**

The WHO partograph has several parts like; i. General description of the women in labour, ii. neonatal part: fetal heart rate, meconium, moulding, iii. Maternal part: cervical dilatation, descent of head & iv. uterine contraction, maternal vital sign and parameters: oxytocin or any other fluid given, v. status of protein, urinary output and keton bodies. All these parts will be filled out through following WHO partograph guides I-IV [10, 38, 39] and IMPAC [10, 12, 38, 39] and also will be used as a guide to operationalize the study for both digital and paper based partograph. An expert programmer or software company will be hired to develop the electronic partograph in line with WHO and IMPAC recommendation. The midwives or nurses who are conducting deliveries in the labour ward will be included and adequate training will be organized for both the groups.

**Training Plan:**

**Paper partograph:**

Expert from OGSB will be consulted for providing training to the nurse or midwives. The existing knowledge on how to use partograph will be assessed for each participant before the start of the training and same assessment will be applied to understand the skill status of partograph usage for each participant. The expert will be trained all the nurses or midwives after getting list from the respected district hospital authority. As the basic of learning are same for both paper & electronic partograph, so, all the selected participants will get same training on partograph. It will be full two days intensive hands on training. A refresher will be organized six months after the first training. As this will be a one year data collection, so there are chances of transfer for midwives to another ward and also posted of new midwives or nurses in labour ward. So, a process will be in place to train those nurses preferably by a local consultant or doctor along with study PI & investigators.

**Electronic partograph:**

In addition of the basic partograph training, the electronic partograph study site participants will also receive training on electronic partograph. The PI, programmer and member from OGSB will conduct this training. It will be a two days training with role play and real life demonstration. Training participants will fill up both the paper-based partograph and electronic partograph at the training period for understanding the similarities and easiness. As mentioned below, electronic partograph application module will be segmented, participants will get training extensively individual module consequently. Participants will also train about the relation and dependency among modules while filling information. About different types of errors and inconsistent scenarios will be introduced to the participants. A junior programmer will be available over mobile phone round-the-clock to provide any instant trouble shooting or feedback and by person for seven days in each month at the field site to oversee the data capture process by electronic partograph tools. Android tablet will be used for implementing the digital partograph study.

**Electronic Partograph design:**

Electronic version of partograph will be a state-of-the-art application that could be accessed through smart phone or tablet pc or computer device. Application's user interface (UI) will be segmented; users will have to concentrate only on a single portion at a time that would lessen the existing complexity of using paper-based partograph. Information architecture of proposed application is given below in Fig: 2

***Fig: 2 Schematic Design for E-Partograph:***

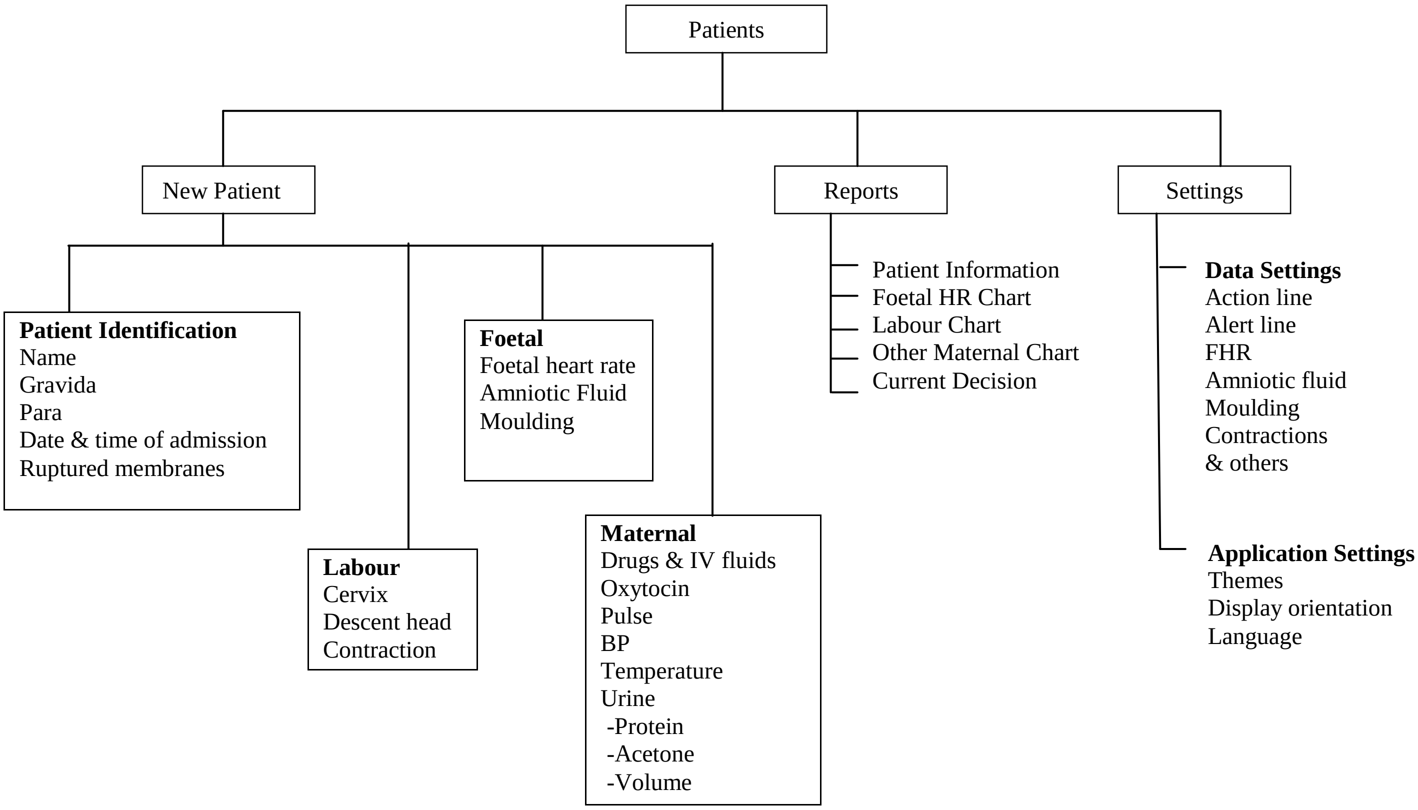


We would like to develop the proposed application's user interface in Android programming language for smart tabs, and in ASP.net with C# language for personal computers.

Application will have options to save the data in local storage and remote central database storage concurrently. Local storage will contain data for limited time duration where remote server will contain the data forever which makes the partograph information searchable in any time and place by this application. The study will use the database server which is already in place in the Centre for Reproductive Health in icddr,b for data storage as a server.

**Definition:**
Prolonged labour is defined as labour extending more than 12 hours and birth asphyxia is defined if APGAR score is less than 7 on either 1st or 5th minute after delivery.

Inclusion and exclusion criteria's have been set as the digital partograph essentially needs to follow the normal and uncomplicated pregnancy where the partograph will avail the opportunity to pass through the usual process of labour.

**Inclusion criteria**
Inclusion criteria for the study will be women with spontaneous labour in the first stage of labour with cervical dilatation in between 4-7 centimeters, singleton pregnancy, gestation of at least 37 completed weeks, cephalic presentations and no additional complications.

**Exclusion criteria**
Exclusion criteria for the study will be women with ante partum hemorrhage, breech presentation, multiple pregnancy, premature labour (before 37 weeks), eclampsia, elective caesarean section and induced labour.

**Implementation Plan:**
To fulfil the specific objectives several different activities will be undertaken during the course of the project which altogether will enable us to address the general objective. The activities are listed below objective wise.

1. To explore the feasibility, acceptability and determining barriers and facilitating factors of introducing digital partograph for its usage by nurse/midwives before and after deploy the crossover design in selected district hospital in Bangladesh:
    a. Activities: Eight to ten Key informant Interview (KII) and twenty in-depth interviews (IDI) will be undertaken to know the feasibility, acceptability and barriers and facilitating factors of the introduced digital partograph. Half of the interviews will be conducted before crossover and rest half will be conducted after applying the crossover design
    b. Participants: Hospital administrators, senior consultant of Obstetric ward, Matron who is in-charge for obstetric ward, Line directors, MNC&AH & programme manager of Reproductive health from Directorate general of Health services will be invited for KII. The midwives and doctors who are involved in conducing deliveries at DH will be invited to take part in IDIs.
    c. Data collection: Data will be collected through qualitative method. Convenient sampling technique will be followed for selecting the participants. A topic guide will be developed at prior of data collection. Qualitative sampling is designed to be information-rich; sampling for the qualitative research will aim to capture the existing situation, challenges and way forward to overcome the problems. It will explore how the new digital partograph affects to increase its usages. It will explore acceptability and feasibility of the new system from providers and recipient point of view. We will conduct KIIs and IDIs at the beginning & end of the each data collection period before and after deploy the crossover design. The qualitative component of the study will lead by the study Co-PI. She is a trained anthropologist and has enough experienced to lead the qualitative study. The recruited Research Assistants will also help her during qualitative data collection. All data will be translated in English.

2. To compare the user rate between paper and digital partograph during labour monitoring process in district hospitals in Bangladesh:

   a. Activities: Will assess the knowledge of the service providers who conducts the deliveries of the two district hospitals and will provide a standard training on WHO modified paper partograph of the two hospitals. Experts from the OGSB will conduct the training. Each training will be conducted for two days. Second Training will be in place before the start of the crossover design. Moreover; the local consultant will be responsible to extend supports if needed for filling out of partograph. In addition of this training the selected hospital's service providers who conduct the deliveries will receive training on digital partograph. This training will be conducted by the programmer, study investigators and experts from OGSB.
   b. Participants: The midwives & doctors who conduct deliveries at district hospitals
   c. Data collection: A structured questionnaire will be developed for paper based partograph and a digital partograph will be developed for android tablet pc. The providers will be extensively oriented on how to plot the readings partograph information at the android pc. At the beginning records for of partograph usages will be extracted from maternity registrars and from patients file records. A test data collection through digital partograph will be conducted in another similar district hospital. In addition one week demonstration will continue at selected hospital for digital partograph to become familiar with the digital partograph by the midwives and doctors. There after the final data collection will start for both the districts hospitals till final sample size achieved for measuring the user rate of the two partograph group. This process will be followed for each before and after deploying the crossover design.
3. To compare the outcome of paper and digital partograph use in respect to the birth asphyxia and prolonged labour rate after introducing digital partograph in selected district hospitals in Bangladesh:
   a. Activity: During the training session of partograph, training to understand birth asphyxia and prolong labour will be in place and this will be guided through a written protocol. The expert will assess the participating midwives and doctors from both hospitals about their understanding on APGAR score and prolong labour. Periodical refreshers training will be in place to guide and understand the knowledge status about APGAR score and prolong labour study participants
   b. Participants: The midwives & doctors who conduct deliveries at district hospitals
   c. Data collection: The rate of birth asphyxia and prolong labour will be extracted from the maternity registrars and patient files of each district hospital at the beginning of the project. A standard protocol developed by OGSB will be in place for measuring APGAR score and prolong labour at each study site for reference. Moreover; one guideline also will be made available for each midwife or doctor who will participate in the study. After each delivery there will be option to record APGAR score and prolong labour both in paper and electronic partograph. During the second training of partograph; APGAR score measurement and prolong labour will also be included. A local expert team combining obstetricians and medical officers will be made to provide the technical support to the study participants throughout the study period. This process will be followed for each before and after deploying the crossover design.

**The following table illustrates the objective specific indicators and proposed methodology and tools for measurement**

| Objective | Indicator | Method | Data collection tools | Implementation Plan |
|---|---|---|---|---|
| 1. To explore the feasibility, acceptability of digital partograph use among health service providers in selected district hospitals in Bangladesh | Providers or users perspective (in terms of acceptability & feasibility) of using digital partograph at district hospital | Qualitative: Key informant interview (KII) and in-depth Interview (IDI) | Semi structured questionnaire | Conduct 4-5 KII of consultant, hospital manager and director or programme manager of MNC&AH and 10 IDIs of midwives or nurses, doctors on before & after applying the crossover design |
| 2. To compare the user rate between paper & digital partograph during labour monitoring process in district hospitals in Bangladesh | Numbers of digital partograph filled out for total deliveries (calculated during sample size calculation) | Record review | • Hospital record<br>• Study MIS | This will be calculated at the baseline and before & after applying the crossover design for each hospital |
| 3. To compare the outcome of digital and paper partograph use in respect to the birth asphyxia (BA) and prolonged labour (PL) rate after introducing digital partograph in selected district hospitals in Bangladesh | i. # of BA occurred for total births<br>ii. # of PL occurred for total births | Record review | • Hospital record<br>• Study MIS | This will be calculated at the baseline, middle of the study and at the end of the study and before & after applying the crossover design for each hospital |
| 4. To determine the barriers and facilitating factors of introducing electronic partograph in district hospital in Bangladesh. | Providers or users perspective (in terms of barriers and facilitating factors) of using digital partograph at district hospital | Qualitative: in-depth Interview (IDI) | Semi structured questionnaire | Conduct 4-5 KII of consultant, hospital manager and director or programme manager of MNC&AH and 10 IDIs of midwives or nurses, doctors and before & after applying the crossover design for each hospital |

**Monitoring plan:**

A Research officer (RO) will be assigned o conduct the in depth interviews and to observe the process of data collection for electronic partograph. This RO will be guided by a qualitative researcher who is also a co-investigator of this study. The RO will also be involved in routine monitoring process of this study. She will check 5% samples of questionnaires with the captured data by both paper and electronic partograph tools. This RO will be posted at the study site. In addition a junior programmer will travel monthly to check the quality of the captured data by both electronic and paper based partograph. The completeness of the partograph will be checked daily basis by the recruited nurses in both the district hospitals. Moreover, PI and the study investigators will travel to the field site periodically to look at the implementation status and quality of collected data. The RO and junior programmer will also provide support to the field staff for any problems if arises. An online system will be in place to look at the completed partograph for each delivery by the hospital manager and obstetric consultant from their office.

## Fig: 3 Monitoring plan:

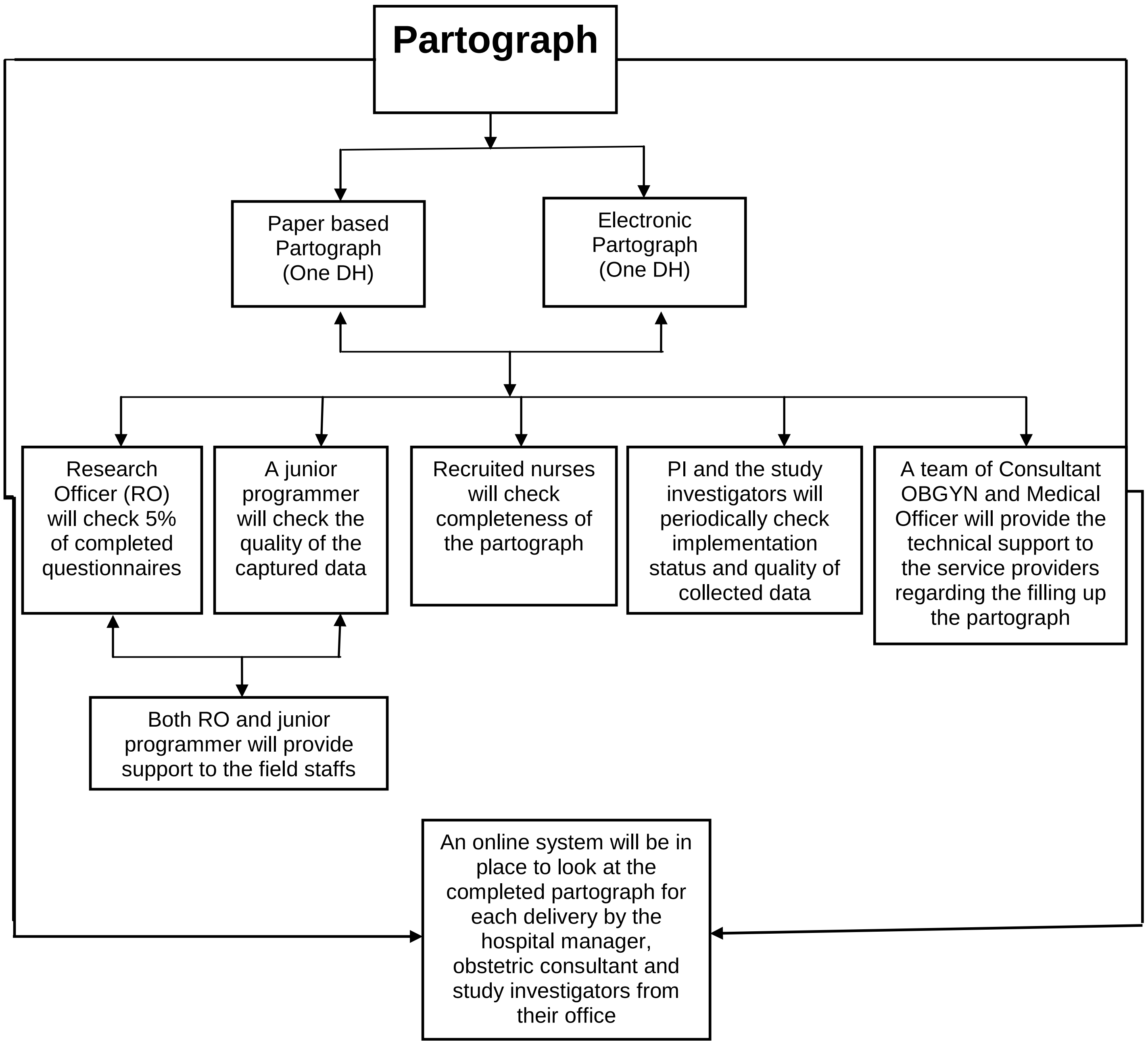

## Timeline:

The study will be completed in 18 months starting from the approval of the RRC and ERC

| List of activities: | 18 months | | | | | | | | |
|---|---|---|---|---|---|---|---|---|---|
| | 1-2 | 3-4 | 5-6 | 7-8 | 9-10 | 11-12 | 13-14 | 15-16 | 17-18 |
| Protocol development, approval & develop application, data tools and training guideline | | | | | | | | | |
| Stakeholders consultation | | | | | | | | | |
| Recruitment &Training of field workers and supervisors | | | | | | | | | |
| Logistic procurement | | | | | | | | | |
| Baseline facility survey &Testing new application | | | | | | | | | |
| Enrolment of the study participant | | | | | | | | | |
| Data collection & follow up | | | | | | | | | |
| Quality control | | | | | | | | | |
| Endline survey, data cleaning and analysis | | | | | | | | | |
| Manuscript writing & Dissemination | | | | | | | | | |

## Sample Size Calculation and Outcome (Primary and Secondary) Variable(s)

Clearly mention your assumptions. List the power and precision desired. Describe the optimal conditions to attain the sample size. Justify the sample size that is deemed sufficient to achieve the specific aims.

## Sample size:

Table 3. Sample size calculations

| **Parameter** | **Current rate (%)** | **Expected prevalence (%)** | **Power and level of significance** | **Minimum sample required** | **Refusal (%)** | **Ultimate sample size** |
|---|---|---|---|---|---|---|
| Use of partograph | 17.1 [40] | 36 | 0.80 and 0.05 | 95 | 10 | 105 |
| Birth asphyxia | 22[41] | 11 | 0.80 and 0.05 | 196 | 10 | 216 |
| Prolonged labour | 7 [42] | 2.8 | 0.80 and 0.05 | 460 | 10 | 506 |

Sampling and sample size calculation:

Sample size calculations will be on the basis of existing prevalence (use) of some parameters such as use of partograph, incidence of birth asphyxia and prolonged labour. Final sample size calculation will also take into account the refusal and design effect. Table 1 shows the sample size calculation on the basis of important parameters those are beneficial due to use of partograph. We had prevalence data on use of partograph and incidence of birth asphyxia and prolonged labour. Sample size exercise shows the study needs to follow deliveries from 105 to 506 (Table 1). Thus the sample size in each district hospital will be 506 mothers who will have deliveries there during the study period. The study will collect described information on half of the sample (253) before applying the crossover design and after applying the crossover design all the information as collected before will be applied for the remaining half (253). Field data collection will be continued for 12 months and the data collectors will try to capture all deliveries till required minimum sample size is achieved. Care will be taken so that equal numbers of deliveries are covered from morning, evening and night shifts.

## Data Analysis

Describe plans for data analysis, including stratification by sex, gender and diversity. Indicate whether data will be analysed by the investigators themselves or by other professionals. Specify what statistical software packages will be used and if the study is blinded, when the code will be opened. For clinical trials, indicate if interim data analysis will be required to determine further course of the study.

### Quantitative data analysis:

Quantitative data will be analyzed using the SPSS statistical software for personal computers. Discrete variables will be expressed as percentages and presented as frequency tables and cross tabulations. $\chi^2$ will be employed as the test of association between proportions of respondents. Statistical significance would be accepted at *p*-values of <0.05. The data collected between the two groups will be analyzed in such a way that a clear comparison could be made between the user rate of different periods (before and after applying the crossover design) after adjusting the potential confounding factors using appropriate multivariate analysis methods such as binary logistic regression.

### Challenges during analysis for crossover design:

Cross over study design was introduced first in a case control study for acute myocardial infarction[43]. In a crossover study the subjects usually passes through both treatment and placebo phases[36]. But five challenges are documented in crossover study design that needs to be taken care during implementation and analysis; (1) Carry over & period effects, (2) treatment sequencing and patient assignment, (3) cross rules and timing measurement, (4) drop outs and (5) statistical analysis and sample size[36, 37].1-3 will be addressed with a 2-3 months paused period and providing same training for two hospitals on both paper and electronic partograph. Four will be addressed through providing training for any new replacement of nurses or doctors of any of the two study site. The fifth one will be addressed through allocating equal number of deliveries in both before and after applying the crossover design and each strata of the study will be analysed separately to know the user rate for each duration and sites.

The adequacy of the partograph itself and of the training programme will be analyzed. The partograph would be critically examined for the accuracy of their completion. In particular, it will be examined whether appropriate action was taken when cervical dilation moved to the right of either the alert line or the action line. If the APGAR score is below 7 either at 1$^{st}$ or 5$^{th}$ minute after birth will be considered as birth asphyxia and if the labour duration is more than 12 hours will also be considered as prolonged labour. These both indicators will be compared in both paper and electronic partograph group.

### Qualitative Data analysis:

Data analysis will be an iterative process, involving concurrent data collection and analysis [19, 25]. The narrative data from the open ended guide line and the responses elicited from the interviews will be compiled in the form of Word text files. The thematic analysis will be conducted through iterative process. Data analysis will include developing of a priori codes. Transcripts will be reading and re-re-reading to find the emerging themes, evaluate strengths and weaknesses of interview techniques, and identify any gaps of data for further exploration. Transcripts will be reviewed carefully, summaries will be developed, and codes will be applied manually. A team approach to analysis will be employed to minimize individual biases.
Intra-coder and inter-coder reliability will be checked. This approach will be applied in every aspects of analysis including coding, with multiple analysts coding the same sections of text to assess inter-coder reliability. A subset of texts will be independently coded to assess inter-coder agreement. Areas of ambiguity will be identified and resolved.

## Data Safety Monitoring Plan (DSMP)

All clinical investigations (research protocols testing biomedical and/or behavioural intervention(s)) should include the Data and Safety Monitoring Plan (DSMP). The purpose of DSMP is to provide a framework for appropriate oversight and monitoring of the conduct of clinical trials to ensure the safety of participants and the validity and integrity of the data. It involves involvement of all investigators in periodic assessments of data quality and timeliness, participant recruitment, accrual and retention, participant risk versus benefit, performance of trial sites, and other factors that can affect study outcome.

**Not applicable**

## Ethical Assurance for Protection of Human rights

Describe the justifications for conducting this research in human participants. If the study needs observations on sick individuals, provide sufficient reasons for using them. Indicate how participants' rights will be protected, and if there would be benefit or risk to each participants of the study. Discuss the ethical issues related to biomedical and social research for employing special procedures, such as invasive procedures in sick children, use of isotopes or any other hazardous materials, or social questionnaires relating to individual privacy. Discuss procedures safeguarding participants from injuries resulting from study procedures and/or interventions, whether physical, financial or social in nature. [Please see Guidelines]

The planned study will be first approved by Research Review Committee (RRC) of International Centre for Diarrhoeal Disease Research, Bangladesh (icddr,b) who will review the technical part of the proposed study. Then we will seek approval of Ethical Review Committee (ERC) of icddr,b who oversee the protection of human rights. Informed consent will be obtained from the hospital administrations, health workers and the mothers in delivery.

## Use of Animals

Describe if and the type and species of animals to be used in the study. Justify with reasons the use of particular animal species in the research and the compliance of the animal ethical guidelines for conducting the proposed procedures.

## Collaborative Arrangements

Describe if this study involves any scientific, administrative, fiscal, or programmatic arrangements with other national or international organizations or individuals. Indicate the nature and extent of collaboration and include a letter of agreement between the applicant or his/her organization and the collaborating organization.

This will be a collaborative agreement between Maternal, Neonatal, child & Adolescent Health (MNC&AH) of DGHS and centre for reproductive health of icddr,b where icddr,b will take the lead role to implement the study and the directorate office of DGHS will provide the administrative support to implement the study.

## Facilities Available

Describe the availability of physical facilities at site of conduction of the study. If applicable, describe the use of Biosafety Level 2 and/or 3 laboratory facilities. For clinical and laboratory-based studies, indicate the provision of hospital and other types of adequate patient care and laboratory support services. Identify the laboratory facilities and major equipment that will be required for the study. For field studies, describe the field area including its size, population, and means of communications plus field management plans specifying gender considerations for community and for research team members.

The server placed in Centre for Reproductive Health, icddr,b will be used to store the captured data for this proposed study.

## Literature Cited

Identify all cited references to published literature in the text by number in parentheses. List all cited references sequentially as they appear in the text. For unpublished references, provide complete information in the text and do not include them in the list of Literature Cited. There is no page limit for this section, however, exercise judgment in assessing the "standard" length.


1. Hogan, M.C., et al., *Maternal mortality for 181 countries, 1980–2008: a systematic analysis of progress towards Millennium Development Goal 5.* The Lancet, 2010. **375**(9726): p. 1609-1623.
2. WHO, U., UNFPA and The World Bank estimates, *Trends in Maternal Mortality: 1990-2012*. 2012.
3. WHO, *Preventing prolonged labour: A practical guide. Maternal health And safe Motherhood programme*. 1994, Division of family health, World Health organization: Geneva.
4. Friedman, E.A., *Primigravid labor: a graphicostatistical analysis.* Obstetrics & Gynecology, 1955. **6**(6): p. 567-589.

5. Philpott, R. and W. Castle, *Cervicographs in the management of labour in primigravidae.* BJOG: An International Journal of Obstetrics & Gynaecology, 1972. **79**(7): p. 592-598.
6. Organization, W.H., *Safe Motherhood Initiative; Nairobi Conference*. 1987.
7. World Health Organization maternal, h. and p. safe motherhood, *World Health Organization partograph in management of labour.* The Lancet, 1994. **343**(8910): p. 1399-1404.
8. Neilson, J., et al., *Obstructed labour Reducing maternal death and disability during pregnancy.* British medical bulletin, 2003. **67**(1): p. 191-204.
9. Dujardin, B., et al., *Value of the alert and action lines on the partogram.* The Lancet, 1992. **339**(8805): p. 1336-1338.
10. *Preventing Prolonged Labour: a practical guide*, in *The Partograph Part I: Principles and Strategy*. 1994, Maternal health and safe Motherhood programme.
11. Mathai, M., *The partograph for the prevention of obstructed labor.* Clinical obstetrics and gynecology, 2009. **52**(2): p. 256-269.
12. Organization, W.H., *IMPAC Integrated management of pregnancy and childbirth. Managing complications in pregnancy and childbirth: a guide for midwives and doctors. Geneva: WHO; 2000.[cited 2013 oct 13]*. 2010.
13. Lennox, C.E. and B.E. Kwast, *The partograph in community obstetrics.* Trop Doct, 1995. **25**(2): p. 56-63.
14. WHO/MCH/88.3, *The Parograph. Guideline for operation research on the application of partograph.* (1988), World Health Organization: Geneva.
15. Kirubakaran, R., Deepak, K.A.,, *A partogram to reduce maternal and infant morbidity and mortality.* Natl Med J India, 1997. **10**(3): p. 152-3.
16. Umezulike, A.C., H.E. Onah, and J.M. Okaro, *Use of the partograph among medical personnel in Enugu, Nigeria.* International journal of gynaecology and obstetrics: the official organ of the International Federation of Gynaecology and Obstetrics, 1999. **65**(2): p. 203-205.
17. Orhue, A., A. Ande, and M. Aziken, *Barriers to the universal application of the portograph for labour managent: A review of the issues and proposed solution.* Tropical Journal of Obstetrics and Gynaecology, 2013. **28**(1): p. 32-42.
18. Pett, C., J. Ruminjo, and K. Levin, *I292 REVITALIZING THE PARTOGRAPH IN LOW RESOURCE SETTINGS: DOES THE EVIDENCE SUPPORT A GLOBAL CALL TO ACTION?* International Journal of Gynecology & Obstetrics, 2012. **119**: p. S235.
19. Vangeenderhuysen, C., P. Renaudin, and M.I. Vall, *The delivery record: a help to make the right decision.* Sante, 2001. **11**(4): p. 259-63.
20. UNFPA, M.o.H.A.f.W.a., *Thematic review of safe motherhood in Bangladesh*. 2004.
21. Blynn, E., *Piloting mHealth: A research scan.* Cambridge, MA: Knowledge Exchange Management Sciences for Health, 2009.
22. Hartung, C., et al. *Open data kit: Tools to build information services for developing regions*. in *Proceedings of the 4th ACM/IEEE International Conference on Information and Communication Technologies and Development*. 2010. ACM.
23. Tamrat, T. and S. Kachnowski, *Special delivery: an analysis of mHealth in maternal and newborn health programs and their outcomes around the world.* Maternal and child health journal, 2012. **16**(5): p. 1092-1101.
24. Parikh, T. and P. Javid, *CAM: A mobile interaction framework for digitizing paper processes in the developing world.* Proc. USIT, 2005.
25. Sherwani, J., et al. *Healthline: Speech-based access to health information by low-literate users*. in *Information and Communication Technologies and Development, 2007. ICTD 2007. International Conference on*. 2007. IEEE.
26. Svoronos, T., et al., *CommCare: Automated Quality Improvement to Strengthen Community-based Health||.* Weston: D-Tree International, 2010.
27. DeRenzi, B., et al. *E-IMCI: Improving pediatric health care in low-income countries*. in *Proceedings of the SIGCHI conference on human factors in computing systems*. 2008. ACM.

28. S Sharmin, M.R., SC Hazra, L Khondker, *Implementation of Partograph and Its Effect on Outcome of Spontaneous Labour at Term.* Bangladesh Medical Journal, 2012 **41**(1).
29. Iqbal Anwar, N.K., and Marge Koblinsky, *Quality of Obstetric Care in Public-sector Facilities and Constraints to Implementing Emergency Obstetric Care Services: Evidence from High- and Low-performing Districts of Bangladesh.* J HEALTH POPUL NUTR, 2009. **27**(2): p. 139-155.
30. Bogren, M.U., *Saving Lives of Mothers and Babies: An Assessment on Nurse-Midwives´ Knowledge in Bangladesh*. 2010.
31. Mahbub Elahi Chowdhury, L.R., Taposh Kumar Biswas, Maksudur Rahman, Shamima Akhter, Ahmed Al-Sabir, *A Needs Assessment Study for Emergency Obstetric and Newborn Care (EmONC) Services in 24 Districts of Bangladesh*. 2014, icddr,b.
32. Evelyn Landry, C.P., Renee Fiorentino, Joseph Ruminjo and Cristina Mattison, *Assessing the quality of record keeping for cesarean deliveries: results from a multicenter retrospective record review in five low-income countries.* BMC Pregnancy and Childbirth, 2014. **14**: p. 139.
33. Aaronson, N.K., *Assessing the quality of life of patients in cancer clinical trials: Common problems and common sense solutions.* European Journal of Cancer, 1992. **28**(8): p. 1304-1307.
34. Erickson, P., R.C. Taeuber, and J. Scott, *Operational aspects of quality-of-life assessment.* Pharmacoeconomics, 1995. **7**(1): p. 39-48.
35. Fitzpatrick R, et al., *Evaluating patient-based outcome measures for use in clinical trials.* Health Technology Assessment, 1998. **2**(14).
36. Louis, T.A., et al., *Crossover and self-controlled designs in clinical research.* The New England journal of medicine, 1984. **310**(1): p. 24-31.
37. Maclure, M., *The case-crossover design: a method for studying transient effects on the risk of acute events.* American journal of epidemiology, 1991. **133**(2): p. 144-153.
38. *Preventing Prolonged Labour: A practical Guide*. 1994, Maternal health and safe Motherhood programme
39. *Preventing Prolonged Labour: A practical Guide* in *The Partograph Part III: Facilitator's Guide*. 1994, Maternal health and safe Motherhood programme.
40. Anwar, I., N. Kalim, and M. Koblinsky, *Quality of obstetric care in public-sector facilities and constraints to implementing emergency obstetric care services: evidence from high- and low-performing districts of Bangladesh* J Health Popul Nutr, 2009. **27**(2): p. 139-55.
41. Islam, M.N., *Situation of Neonatal Health in Bangladesh.* The Orion, 2000. **6**.
42. Rather, S.Y., et al., *Use of condom to control intractable PPH.* JK science, 2010. **12**(3).
43. Muller, J.E., et al., *Circadian variation in the frequency of onset of acute myocardial infarction.* New England Journal of Medicine, 1985. **313**(21): p. 1315-1322.

# (Annexure: 3)

# Written consent form (Quantitative) for Service providers

| Protocol No. PR-14011 | Version No. 3.00 | Date: 05-01-2015 |
|---|---|---|

**Protocol Title:** Electronic partograph: A way of improving partograph use during labour monitoring process in selected district hospitals in Bangladesh

**Investigator's name:** Dr. Aminur Rahman
**Organization:** icddr,b

## Purpose of the research

**Background** (brief introduction of the issue and the need for/ importance of the research)

Good [morning/afternoon]. My name is [your name] working for icddr,b in a research study title "Electronic partograph: A way of improving partograph use during labour monitoring process in selected district hospitals in Bangladesh". We will just observe and record information through using paper/electronic partograph by you. This information will help us to estimate the user rate of partograph that is important to prevent prolonged labour, birth asphyxia and also promote timely decisions during labour. The results of this study will help to reduce maternal and perinatal mortality & morbidity in Bangladesh through increasing the rate of partograph use.

## Why invited to participate in the study?

As you are a service provider posted in this obstetric unit of this hospital, you will be under observation during the labour period of your patient. We need to collect the information which is required for partograph. This is why we are inviting you to help us by participating in this study.

## Methods and procedures [What is expected from the participants of the research study?]

If you agree to our proposal of including you in the study, you might expect the followings:

We will observe your partograph documentation process when you fill it out during labour monitoring process of a birthing woman. We assure you the collected data will not be used for any other purpose. Your participation is voluntary in this study.

## Risk and benefits

As we will just observe you and record information regarding your partograph documentation process, there will not be any risk to you if you participate in the study. There are no direct benefits to you. But, if you are agree, you will be asked to participate in the research study to improve the partograph use which ultimately will improve the maternal and perinatal health in public hospitals of Bangladesh.

## Privacy, anonymity and confidentiality

Please be assured that the information will be recorded regarding your services will be treated in a confidential manner. Only physicians, investigators of this study, and members of the Ethical Review Committee of icddr,b will have access to the information collected. Many participants will be included in this study and your information will be combined with theirs to be reviewed and reported as a group. You will not be personally identified in any study report. We will use only a number to identify you. If you have any question you can ask me without any hesitation. I am ready to answer all your questions.

**Future use of information**

The information captured through paper/electronic partograph is aimed at improving quality of care and reducing mortality and morbidly during and around delivery. We assure you that privacy, anonymity, and confidentiality will be maintained. Your name will not be used in any report.

**Right not to participate and withdraw**

Your participation is voluntary. You have the right to stop us to observe you during the services provided by you to the mother at any time.

**Principle of compensation**

As mentioned earlier, your participation in this study is completely voluntary and you will not get any payment for participating in this study.

**Answering your questions/ Contact persons**

If you have any question, you can ask me any time. If you have additional questions about the study, you may contact: Dr.Aminur Rahman, Centre for Reproductive Health, icddr,b, Mohakhali, Dhaka 1212. Phone: 9840523-32 # 2200. If you have questions about your rights as a participant of a research study, or if you think some harm has been done to you because of the participation, you may contact: M. A. Salam Khan, IRB Secretariat, phone: 9886498 or PABX 9840523-32 ext. 3206.

If you agree to our proposal of enrolling you/your patient in our study, please indicate that by putting your signature or your left thumb impression at the specified space below-

Thank you for your cooperation

______________________________________ ____________________

Signature or left thumb impression of participant Date

______________________________________ ____________________

Signature or left thumb impression of the witness Date

______________________________________ ____________________

Signature of the PI or his/her representative Date

(NOTE: In case of representative of the PI, she/he shall put her/his full name and designation and then sign)

(Name and contact phone of IRB Secretariat, RA, M. A. Salam Khan, Phone No: 9886498 or PABX 8860523-32 Extension. 3206).

(Annexure: 5)

# Assent Form for Mothers (Quantitative)
# (Below 18 years)

| Protocol No. PR-14011 | Version No. 3.00 | Date: 05-01-2015 |
|---|---|---|

**Protocol Title:** Electronic partograph: A way of improving partograph use during labour monitoring process in selected district hospitals in Bangladesh

**Investigator's name:** Dr. Aminur Rahman
**Organization:** icddr,b

## Purpose of the research

**Background** (brief introduction of the issue and the need for/ importance of the research)

Good [morning/afternoon]. My name is [your name] working for icddr,b in a research study title "Electronic partograph: A way of improving partograph use during labour monitoring process in selected district hospitals in Bangladesh". We will just observe and record information through using paper/electronic partograph. This information will help us to estimate the user rate of partograph that is important to prevent prolonged labour, birth asphyxia and also promote timely decisions during labour. The results of this study will help to reduce maternal and perinatal mortality & morbidity in Bangladesh through increasing the rate of partograph use.

## Why invited to participate in the study?

As you are admitted in this obstetric unit of this hospital, you will be under observation during the labour. We need to collect the information which is required for partograph. This is why we are inviting you to help us by participating in this study.

## Methods and procedures [What is expected from the participants of the research study?]

If you agree to our proposal of including you in the study, you might expect the followings:
We would enrol you in this research study during your labour process. Your labour process will be monitored through paper/electronic partograph by nurses or doctors that will help them to understand whether your labour is progressing to the right direction or not. We assure you that the collected data will not be used for any other purpose. The participation is voluntary.

## Risk and benefits

As we will just observe you and will record information regarding your labour process, there will not be any risk to you if you participate in the study. There are no direct benefits to you. But, if you agree, you will be asked to participate in the research study to improve the partograph use which ultimately will improve the maternal and perinatal health in public hospitals of Bangladesh.

## Privacy, anonymity and confidentiality

Please be assured that the information will be recorded regarding your labour process will be treated in a confidential manner. Only physicians, investigators of this study, and members of the Ethical Review Committee (ERC) of icddr,b will have access to the information collected. Many participants will be included in this study and your information will be combined with theirs to be reviewed and reported as a group. You will not be personally identified in any study report. We will use only a number to identify you. If you have any question you can ask me without any hesitation. I am ready to answer all your questions.

## Future use of information

The information captured through paper/electronic partograph is aimed at improving quality of care and reducing mortality and morbidly during and around delivery. We assure you that privacy, anonymity, and confidentiality will be maintained. Your name will not be used in any report.

**Right not to participate and withdraw**
Your participation is voluntary. You have the right to stop us to observe you at any time. Even if you withdraw from the study, you will receive the same quality of medical care for which you have been admitted in this hospital.

**Principle of compensation**
As mentioned earlier, your participation in this study is completely voluntary and you will not get any payment for participating in this study.

**Answering your questions/ Contact persons**
If you have any question, you can ask me any time. If you have additional questions about the study, you may contact: Dr.Aminur Rahman, Centre for Reproductive Health, icddr,b, Mohakhali, Dhaka 1212. Phone: 9840523-32 # 2200. If you have questions about your rights as a participant of a research study, or if you think some harm has been done to you because of the participation, you may contact: M. A. Salam Khan, IRB Secretariat, phone: 9886498 or PABX 9840523-32 ext. 3206.

If you agree to our proposal of enrolling you/your patient in our study, please indicate that by putting your signature or your left thumb impression at the specified space below-

Thank you for your cooperation

_________________________________ ________________
Signature or left thumb impression of participant Date

_________________________________ ________________
Signature or left thumb impression of
Parent/ Guardian/ Attendant Date

_________________________________ ________________
Signature or left thumb impression of the witness Date

_________________________________ ________________
Signature of the PI or his/her representative Date

(NOTE: In case of representative of the PI, she/he shall put her/his full name and designation and then sign)

(Name and contact phone of IRB Secretariat, RA, M. A. Salam Khan, Phone No: 9886498 or PABX 8860523-32 Extension. 3206).

# (Annexure: 7)

## Voluntary Consent Form (Qualitative)

(For Doctors, Matron/Nurses, Hospital managers)

| Protocol No. PR-14011 | Version No. 1.00 | Date: 30-11-2014 |
|---|---|---|

**Protocol Title:** Electronic partograph: A way of improving partograph use during labour monitoring process in selected district hospitals in Bangladesh

**Investigator's name:** Dr. Aminur Rahman

**Organization:** icddr,b

**Background** (brief introduction of the issue and the need for/ importance of the research)
Asslamualaikum/Adab/Namoskar. My name is [your name] working for icddr,b in a research study titled "Electronic partograph: A way of improving partograph use during labour monitoring process in selected district hospitals in Bangladesh" We will just record information on your views and opinions regarding electronic partograph. This information will help us to explore the feasibility, acceptability of electronic partograph use among health service providers in selected district hospitals in Bangladesh. Partograph is very important to prevent prolonged labour, birth asphyxia and also promote timely decisions during labour. The results of this study will help to reduce maternal and perinatal mortality & morbidity in Bangladesh through increasing the rate of partograph use.

**Why invited to participate in the study?**
We are inviting you to participate in this study for an in-depth interview because your valuable opinion will help us to explore the existing situation and the feasibility of the electronic partograph tool. As part of this study we will also include views and opinions from many people like you.

**Methods and procedures [What is expected from the participants of the research study?]**
If you agree to participate in the study, you might expect the followings:
We will take your interview and it may take about 40-45 minutes. We will ask you some questions regarding the paper/electronic partograph to know your opinion.

**Risk and benefits**
There are no risks to you if you attend the discussion. Nothing harmful will come from it. There are no direct benefits to you. If you are agree, you will be asked to participate in the research study to improve the partograph use which ultimately will improve the maternal and perinatal health in public hospitals of Bangladesh.

**Privacy, anonymity and confidentiality**
Although during the discussion I will write down some notes; you can fully rely on me to keep confidential your identity and the information you provide. I would like to record our conversation so that I can remember what we talked about. Is it ok if I record our conversation? [Wait for the respondent to answer 'Yes' or 'No'. If 'Yes', continue to read. If 'No', ask the respondent if we can continue without recording the session.] Is this a good time for you to talk? [Wait for the answer 'Yes' or 'No'. If 'Yes', continue to read. If 'No', ask the respondent if there is a better time and then re-schedule the discussion.] The papers containing the information will remain with us at icddr, b in a locked cabinet and no one except the people involved with this research will be able to see the information. No other person (except study investigators and ERC members) will be able to look at the interview notes and your name will not be on the paper with the notes. We will use only a number to identify you. If you have any question you can ask me without hesitation. I am ready to answer all your questions.

## Future use of information

The information we will capture through the interview is aimed at improving quality of care and reducing mortality and morbidly during and around delivery. We assure you that privacy, anonymity, and confidentiality will be maintained.

**Right not to participate and withdraw**

Your participation and cooperation is entirely voluntary. It is your decision. Also you have the right not to answer any questions you do not want to. You may also withdraw yourself from the study at any time.

**Principle of compensation**

As mentioned earlier, your participation in this study is completely voluntary and you will not get any payment for participating in this study.

**Answering your questions/ Contact persons**

If you have any question, you can ask me any time. If you have additional questions about the study, you may contact: Dr.Aminur Rahman, Centre for Reproductive Health, icddr,b, Mohakhali, Dhaka 1212. Phone: 9840523-32 # 2200. If you have questions about your rights as a participant of a research study, or if you think some harm has been done to you because of the participation, you may contact: M. A. Salam Khan, IRB Secretariat, phone: 9886498 or PABX 9840523-32 ext. 3206.

If you agree to our proposal of enrolling you/your patient in our study, please indicate that by putting your signature or your left thumb impression at the specified space below-

Thank you for your cooperation

_______________________________________ ____________________

Signature or left thumb impression of participant Date

_______________________________________ ____________________

Signature or left thumb impression of the witness Date

_______________________________________ ___________________

Signature of the PI or his/her representative Date

(NOTE: In case of representative of the PI, she/he shall put her/his full name and designation and then sign)

(Name and contact phone of IRB Secretariat, RA, M. A. Salam Khan, Phone No: 9886498 or PABX 8860523-32 Extension. 3206).

# PARTOGRAPH

Registration No.____________ Name (Last, First)________________________________ Age_____

Date______________Parity/Gravida_____/______LMP_______EDD________Gestation (wks)_____

ROM (Time, Date)____/________ Labour Duration (Hrs)_____ Facility/Clinic Name________________

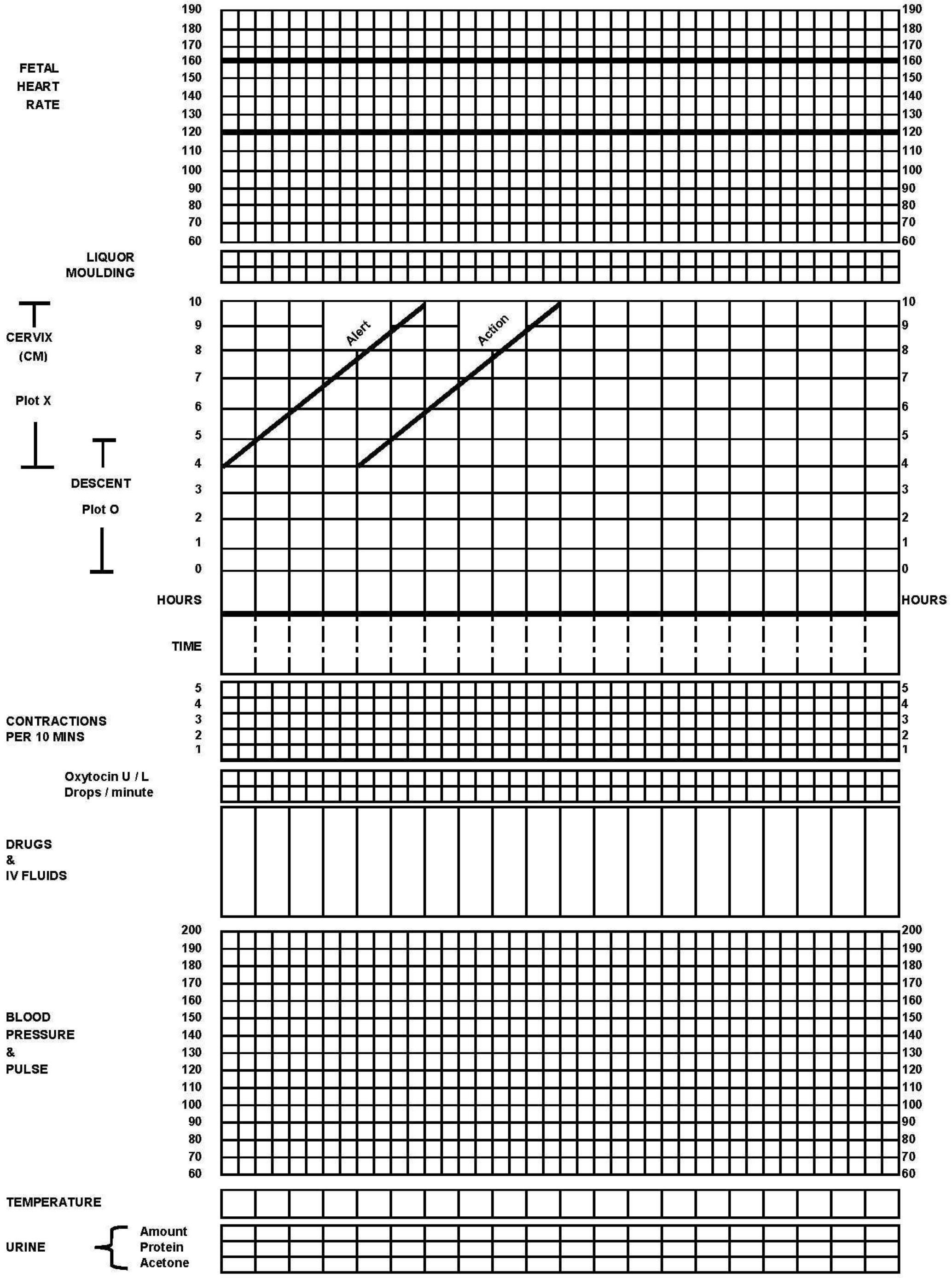

(Annexure: 10)

# Partograph Questionnaire

General Description:

1. Registration #
2. Name:
3. Age:
4. Date of admission:
5. Parity
6. Gravida:
7. LMP:
8. EDD:
9. Gestational weeks:
10. Rupture of membrane:
    a. Date:
    b. Time:
11. Labour Duration:
12. Clinic/facility name:
13. 1st Measurement date and time:
    a. Fetal hear rate: b/m
    b. Liquor: intact=1, clear=2, meconium:3, blood stain:4, other:5
    c. Moulding: No gap=0, ‘+’ =1, ‘++’ =2, ‘+++’ =3, Overlapping=5
    d. Cervical dilatation: ………… Cm
    e. Plotting: left of alert line:1, On alert line=2, Right of alert line=3, Over action line=4, Right to action line=5
    f. Head descent: 0/0=0, 1/5=1, 2/5=2, 3/5=3, 4/5=4, 5/5=5
    g. Duration of uterine contraction per 10 minute: sec
    h. Number of uterine contraction per 10 minute:
    i. Inj. oxytocin given: Yes=1, No=2
    j. Quantity of oxytocin=
    k. Flow: drop/min
    l. Drugs:
    m. Fluid:
    n. Blood pressure: i. Systolic: mm of Hg, ii. Diastolic: mm of Hg
    o. Pulse: b/m
    p. Temperature: $^{0}$F
    q. Urine output: ml
    r. Protein:
    s. Acetone:
14. 2nd Measurement date and time:
    a. Fetal heart rate: b/m
    b. Liquor: intact=1, clear=2, meconium:3, blood stain:4, other:5
    c. Moulding: No gap=0, ‘+’ =1, ‘++’ =2, ‘+++’ =3, Overlapping=5

d. Cervical dilatation: …………. Cm
e. Plotting: left of alert line:1, On alert line=2, Right of alert line=3, Over action line=4, Right to action line=5
f. Head descent: 0/0=0, 1/5=1, 2/5=2, 3/5=3, 4/5=4, 5/5=5
g. Duration of uterine contraction per 10 minute: sec
h. Number of uterine contraction per 10 minute:
i. Inj oxytocin given: Yes=1, No=2
j. Quantity of oxytocin=
k. Flow: drop/min
l. Drugs:
m. Fluid:
n. Blood pressure: i. Systolic: mm of Hg, ii. Diastolic: mm of Hg
o. Pulse: b/m
p. Temperature: $^0$F
q. Urine output: ml
r. Protein:
s. Acetone:

15. 3rd measurement date and time:
    a. Fetal heart rate: b/m
    b. Liquor: intact=1, clear=2, meconium:3, blood stain:4, other:5
    c. Moulding: No gap=0, '+' =1, '++' =2, '+++' =3, Overlapping=5
    d. Cervical dilatation: …………. Cm
    e. Plotting: left of alert line:1, On alert line=2, Right of alert line=3, Over action line=4, Right to action line=5
    f. Head descent: 0/0=0, 1/5=1, 2/5=2, 3/5=3, 4/5=4, 5/5=5
    g. Duration of uterine contraction per 10 minute: sec
    h. Number of uterine contraction per 10 minute:
    i. Inj oxytocin given: Yes=1, No=2
    j. Quantity of oxytocin=
    k. Flow: drop/min
    l. Drugs:
    m. Fluid:
    n. Blood pressure: Systolic: mm of Hg, Diastolic: mm of Hg
    o. Pulse: b/m
    p. Temperature: $^0$F
    q. Urine output: ml
    r. Protein:
    s. Acetone:
16. 4th Measurement date and time:
    a. Fetal hear rate: b/m
    b. Liquor: intact=1, clear=2, meconium:3, blood stain:4, other:5
    c. Moulding: No gap=0, '+' =1, '++' =2, '+++' =3, Overlapping=5
    d. Cervical dilatation: …………. Cm

e. Plotting: left of alert line:1, On alert line=2, Right of alert line=3, Over action line=4, Right to action line=5
f. Head descent: 0/0=0, 1/5=1, 2/5=2, 3/5=3, 4/5=4, 5/5=5
g. Duration of uterine contraction per 10 minute: sec
h. Number of uterine contraction per 10 minute:
i. Inj oxytocin given: Yes=1, No=2
j. Quantity of oxytocin=
k. Flow: drop/min
l. Drugs:
m. Fluid:
n. Blood pressure: i. Systolic: mm of Hg, ii. Diastolic: mm of Hg
o. Pulse: b/m
p. Temperature: $^{0}$F
q. Urine output: ml
r. Protein:
s. Acetone:

17. 5th Measurement date and time:
a. Fetal hear rate: b/m
b. Liquor: intact=1, clear=2, meconium:3, blood stain:4, other:5
c. Moulding: No gap=0, '+' =1, '++' =2, '+++' =3, Overlapping=5
d. Cervical dilatation: …………. Cm
e. Plotting: left of alert line:1, On alert line=2, Right of alert line=3, Over action line=4, Right to action line=5
f. Head descent: 0/0=0, 1/5=1, 2/5=2, 3/5=3, 4/5=4, 5/5=5
g. Duration of uterine contraction per 10 minute: sec
h. Number of uterine contraction per 10 minute:
i. Inj oxytocin given: Yes=1, No=2
j. Quantity of oxytocin=
k. Flow: drop/min
l. Drugs:
m. Fluid:
n. Blood pressure: i. Systolic: mm of Hg, ii. Diastolic: mm of Hg
o. Pulse: b/m
p. Temperature: $^{0}$F
q. Urine output: ml
r. Protein:
s. Acetone:

# (Annexure: 11)

<u>Interview Guideline</u>
<u>Key Informant Interviews</u>

Name:
Age:
Educational background:
Place of interview:
Date of Interview:

**1.** Identify the key informant’s awareness of about the tool
   a. Have you heard about electronic partograph?
   b. If yes, what did you hear about it?
   c. How did you hear about it?

**2.** In the context of Bangladesh, explore key informant’s experience, barriers and feasibility of implementing the electronic partograph in health facility?
   a. What is your experience about the current use of this electronic partograph?
   b. Why did you decide to use this electronic partograph?
   c. Why it is useful?
   d. What are the advantages of the using the electronic partograph?
   e. What the disadvantages of using the electronic partograph?

**3.** Explore the future strategies and motivation
   a. How this electronic partograph can be established in your health system?
   b. What were the main motivating factors that have influenced you and your team to implement the electronic partograph in your system?
   c. Please tell me more about your future strategy to continue the program?
   d. What are challenges you have faced to during using the tool?
   e. What are challenges you have faced to during using the tool?

**4.** Can tell me about the cost of implementing the new tool in health facilities in Bangladesh?

(Annexure: 13)

## Guideline for In depth informant Interview:

Name:
Age:
Educational background:
Place of interview:
Date of Interview:

1. Introduction: Identify the informant’s awareness of about the tool
   i) Have you heard of digital partograph?
   ii) If yes, what did you hear about it?
   iii) How did you hear about it?
2. Explore informant’s experience and barriers about tool
   i) What is your experience about the tool?
   ii) Why did you decide to use this tool
   iii) What are they good for?
   iv) What are the advantages of the using the tool?
   v) What the disadvantages of using the tool?
3. Explore the future strategies and motivation
   i) How have established the system of digital partograph in your health system?
   ii) What were main motivating factors that influence your team to implement the tool in your system?
4. Please tell me more about your future strategy to continue the program?
5. What are challenges you have faced to during using the tool?
6. How have you overcome the challenges?
7. Can tell me about the workload you and your staff faced for using the new tool?

# Check-List

## Check-list for Submission of Research Protocol
## For Consideration of the Research Review Committee (RRC)
## [Please check all appropriate boxes]

1. Has the proposal been reviewed, discussed and cleared by all listed investigators?

   ☒ Yes ☐ No

   If the response is No, please clarify the reasons:

2. Has the proposal been peer-reviewed externally?

   ☒ Yes ☐ No ☐ External Review Exempted

   If the response is 'No' or "External Review Exempted", please explain the reasons:

   If the response is "Yes", please indicate if all of their comments have been addressed?

   ☒ Yes (please attach)

   ☐ No (please indicate reason(s)):

3. Has the budget been reviewed and approved by icddr,b's Finance?

   ☒ Yes ☐ No (reason): ____________________

4. Has the Ethics Certificate(s) been attached with the Protocol?

   ☒ Yes ☐ No

   If the answer is 'No', please explain the reasons:

______________________________
Signature of the Principal Investigator

30.11.2014
Date

**Gender Analysis Matrix:**

**Study Title:** Electronic partograph: A way of improving partograph use during labour monitoring process in selected district hospitals in Bangladesh

| In relation with introducing electronic partograph | Are there sex difference | How do biological difference b/w women & men influence their: | How do the different roles & activities of men & women affect their | How do gender norms & value affect men & women's | How do access to & control over resources affect men & women's |
|---|---|---|---|---|---|
| Vulnerability Incidence Prevalence | This tool is only for woman | No difference | General people have no idea about partograph | In periphery the paper based partograph user rate is too low | |
| Health seeking behavior | No difference | This device will be used only for women | If the digital partograph increase the quality of care the women will rush to the hospital for deliveries | This digital system can make the norm equal for both | Women have less access and control on monetary issue of the family for which they can't take their own decision for health seeking |
| Ability to access health services | This tools is only for woman | If women can reach to the hospital this digital device will make no difference in terms of getting access | If man is aware about the use of partograph they will be interested more to bring their wife to the facility for conducting deliveries | As women are not the main decision maker on care seeking during delivery in our country they might not get permission to come to the hospital. | As this system is not available yet so final comments cannot be made yet |
| Experience with health services & health providers | Mostly female conduct normal deliveries in Bangladeshi hospitals | In obs ward men are less allowed | | As it is new the man & woman may not be aware whether this digital device is being used or not during delivery | In our society usually men have enough resources. sometimes this make them confident to get services |
| Preventive & treatment option, response to Rx or Rehabilitation | Preventive measure may very if this tool is not used during labour | In emergency cases this tool may not be used | | Values/norm may affect for permitting to use the digital partograph | |
| Outcome of health problem | Rx difference may observe among women if though digital partograph is available for use | It depends when the woman arrives t hospital for delivery | It depends of women's in her family and role of service providers | Equal for both in terms of digital information | Income generating women gets more priority for receiving the appropriate treatment |
| Consequences (economic& | Delayed or mal treatment could be catastrophic | As women are more anemic so they must go | As pregnancy is expected and desirable in our | | |

| In relation with introducing electronic partograph | Are there sex difference | How do biological difference b/w women & men influence their: | How do the different roles & activities of men & women affect their | How do gender norms & value affect men & women's | How do access to & control over resources affect men & women's |
|---|---|---|---|---|---|
| social including attitudinal) | for women. But this technology might be useful for them in rural setting | under partograph monitoring to avoid further complication | society so women receive more care. But it depends also on her status in her family | | |
